# Development and Validation of a Large Language Model Case Identification Strategy for Eosinophilic Esophagitis

**DOI:** 10.1016/j.gastha.2026.100971

**Published:** 2026-04-16

**Authors:** Corey J. Ketchem, Uğurcan Vurgun, Agnes Wang, Sunil Thomas, Ashley Batugo, John E. Pandolfino, Gary W. Falk, Kristle L. Lynch, Evan S. Dellon, Danielle L. Mowery, James D. Lewis

**Affiliations:** 1 Division of Gastroenterology and Hepatology, Department of Medicine, Perelman School of Medicine, Hospital of the University of Pennsylvania, Philadelphia, Pennsylvania; 2 Division of Gastroenterology and Hepatology, Department of Medicine, Kenneth C. Griffin Esophageal Center of Northwestern Medicine, Feinberg School of Medicine, Northwestern University, Chicago, Illinois; 3 Department of Biostatistics, Epidemiology, & Informatics, University of Pennsylvania, Philadelphia, Pennsylvania; 4 Department of Computer and Information Science, School of Engineering & Applied Sciences, University of Pennsylvania, Philadelphia, Pennsylvania; 5 Institute for Biomedical Informatics, Perelman School of Medicine, University of Pennsylvania, Philadelphia, Pennsylvania; 6 Center for Esophageal Diseases and Swallowing, Division of Gastroenterology and Hepatology, Department of Medicine, University of North Carolina School of Medicine, Chapel Hill, North Carolina; 7 Division of Gastroenterology and Hepatology, Department of Medicine, Center for Gastrointestinal Biology and Disease, University of North Carolina School of Medicine, Chapel Hill, North Carolina

**Keywords:** Eosinophilic Esophagitis, Natural Language Processing, Artificial Intelligence, Electronic Health Records, Algorithms

## Abstract

**Background and Aims:**

Epidemiologic research in eosinophilic esophagitis (EoE) is limited by the accuracy and efficiency of case identification algorithms. We aimed to evaluate rule-based natural language processing (RB-NLP) and large language model–based natural language processing (LLM-NLP) pipelines for identifying EoE diagnoses and features from unstructured text.

**Methods:**

We identified gastrointestinal pathology reports with any mention of “eosinophil” paired with gastroenterology clinic notes. Three hundred randomly selected patients were divided into training (n = 200, 56 with EoE) and testing (n = 100, 36 with EoE) sets. Manual chart review was the reference standard. RB-NLP used spaCy with medspaCy’s clinical components; LLM-NLP prompts were developed through iterative human-in-the-loop refinement. In the validation set, we compared International Classification of Diseases (ICD) codes, RB-NLP, and LLM-NLP against the reference standard using sensitivity (recall), positive predictive value (precision), and F1 score.

**Results:**

In the validation set, ICD codes alone had a sensitivity 0.86 (95% confidence interval [CI]: 0.75–0.97), a positive predictive value of 0.97 (95% CI: 0.91–1.0), and an F1 value of 0.91 (95% CI: 0.84–1.0). Combining ICD and LLM-assigned diagnosis yielded a 3-point improvement in F1 score (95% CI: −0.01 to 0.07; *P* = .2) compared to ICD alone. In a larger cohort (n = 580), the LLM + ICD approach identified the most EoE cases (n = 203) and captured 15% of cases missed by ICD codes. Clinical characteristics varied depending on the case identification strategy used.

**Conclusion:**

Combining LLM-NLP with a single ICD code reduced false negatives and modestly improved the F1 score compared to either method alone. This may represent a scalable approach to enhance EoE case identification in real-world data.

## Introduction

Eosinophilic esophagitis (EoE) is a chronic, allergen-mediated inflammatory disease that negatively impacts patients’ quality of life and often follows a progressive course.^1^^,^^2^ The incidence and prevalence of EoE have increased significantly over the past 15 years, leading to increased health-care cost and EoE-related emergency department visits.^3^^,^^4^ These patterns highlight the need for high-quality research studies to address key knowledge gaps and inform strategies to mitigate the rising disease burden.^5^ However, large-scale epidemiologic research for EoE is hampered by suboptimal accuracy of International Classification of Diseases (ICD) administrative codes, which suffer from both high false-positive and false-negative rates.^6^, ^7^, ^8^ Although case identification algorithms requiring multiple ICD codes for EoE reduce false positives, such algorithms may exclude true cases, further impacting epidemiologic studies.

In clinical practice, a diagnosis of EoE requires integration of symptoms and histopathologic findings, much of which is documented in unstructured electronic health record (EHR) text. While manual chart review can be utilized for case identification, it is resource intensive, time consuming, and impractical at scale. Clinical natural language processing (NLP)—a field at the intersection of biomedicine, computer science, and artificial intelligence—offers a promising solution by enabling the extraction of key clinical concepts from unstructured EHR data. The traditional rule-based natural language processing (RB-NLP) approach has previously been evaluated in several gastrointestinal disease states.^9^, ^10^, ^11^ However, recent advances in large language models (LLMs) have expanded NLP capabilities by enabling flexible, high-performance language understanding through natural language prompts, allowing information extraction without extensive programming or domain-specific linguistic expertise. This presents opportunities to standardize data extraction, minimize manual chart review, and process larger data volumes, with the goal of improving data quality and lowering cost.^12^^,^^13^ As such, LLMs may prove valuable for diseases such as EoE, where diagnosis relies on integrating information from multiple sources.

We aimed to evaluate EoE case identification pipelines using RB-NLP and large language model–based natural language processing (LLM-NLP), benchmarked against a manually annotated reference standard. We hypothesized that LLM-NLP would improve recall (sensitivity) and precision (positive predictive value [PPV]) for identifying EoE cases and increase case yield when applied to a separate cohort.

## Methods

### Cohort Construction and Reference Standard

**Figure 1:**
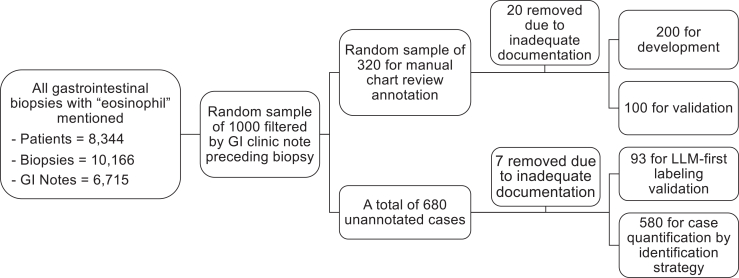
Cohort construction and annotation workflow for natural language processing pipeline.

To develop the cohort, we queried Penn Medicine patients with clinical encounters from 2008 through April 2024, with patients initially identified by surgical pathology procedure orders (Figure 1). For each order, all associated “Final Diagnosis” unstructured text was aggregated, and records containing the term “eosinophil” were flagged regardless of the context; therefore, specimens with “no eosinophils” were also included. These were further filtered to include samples from relevant gastrointestinal anatomic sites (eg, esophagus, stomach, duodenum, colon, small intestine). Gastroenterology clinical encounters 1 year before or after the pathology were identified, and the full unstructured clinical text from progress notes was aggregated. “Inadequate documentation” was defined as clinic note text that lacked sufficient symptom information to assess for EoE diagnostic criteria and were removed during manual review for development and validation. All data were deidentified using Philter, an open source deidentification tool.^14^ Demographics and ICD codes for EoE (all converted to ICD-10, K20.0) were collected from all available time points, while age was at first biopsy.

Diagnostic classification for both the reference standard (manual review by C.J.K.) and NLP-based approaches followed a predefined decision algorithm. Patients were classified as having EoE if either of the following criteria were met: (1) a prior diagnosis of EoE was identified in the clinic note and not negated; or (2) pathology features extracted from the pathology report met histologic criteria for EoE (≥15 eosinophils per high-power field) in conjunction with esophageal dysfunction symptoms extracted from the clinic note.

For both NLP methods used, we included patients with at least 1 associated clinical note between 1 and 60 days prior to the corresponding biopsy date (Supplementary Methods). This window was chosen to balance capturing clinically relevant symptoms that reflected the prebiopsy context while minimizing inclusion of outdated or postdiagnostic documentation that could introduce bias. Subjects were randomly allocated into development (n = 200; 56 with EoE) and validation (n = 100; 36 with EoE) sets. Assuming a PPV of 80%, the sample sizes yielded 95% confidence intervals (CIs) with margins of error ranging from ±9% to ±11%.

### Algorithms for EoE Case Identification

We examined 3 different approaches to EoE case identification. The ICD-only identification strategy was defined as having ever received at least 1 EoE-related ICD code during the available observation period.

The RB-NLP pipeline analyzed the unstructured pathology and clinic notes using spaCy, with medspaCy’s clinical components enabled (Supplementary Methods).^15^ Sentence segmentation was performed using a custom boundary component followed by spaCy’s sentencizer to improve parsing of structured clinical text. We implemented curated TargetRule definitions with medSpaCy’s TargetMatcher to identify key EoE-related concepts (Supplementary Table 1). Classification as a positive diagnosis of EoE was the same as previously mentioned, termed “RB-NLP diagnosis.” The “RB + ICD diagnosis” classified patients as positive if they ever had an EoE-related ICD code and/or met RB-NLP diagnosis. All features were evaluated at the document level, and no machine learning classifier was used. All features and diagnoses were compared to the reference standard.

The LLM-NLP pipeline utilized OpenAI GPT-4-turbo-128k, deployed using the secure and Health Insurance Portability and Accountability Act-compliant Microsoft Azure Databricks infrastructure within Penn Medicine’s tenant (Supplementary Methods). We implemented a structured prompt engineering approach that instructed the model to extract diagnostic variables that were used to assign an EoE diagnosis, which were then mapped to diagnosis using a prespecified rules-based decision layer. Supplementary Table 1 summarizes the primary clinical concepts extracted from the notes, including dysphagia, food impaction, reflux symptoms, eosinophil presence, eosinophil count per high-power field, and prior history of EoE, among others. This framework defined EoE based on either a documented history of EoE or concordant symptom and pathology findings (“LLM-features diagnosis”). In parallel, the model was prompted to independently assign an EoE diagnosis based on the clinic and pathology text alone, without reliance on extracted features (“LLM-assigned diagnosis”). Finally, the “LLM + ICD diagnosis” approach classified patients as positive if they ever had an EoE-related ICD code and/or met the LLM-assigned diagnosis criteria. During development, a human-in-the-loop approach was used for iterative prompt refinement (Supplementary Figure 1; Supplementary Methods).^16^ Once finalized, the prompt was locked, and performance was evaluated in an independent validation cohort.

### Statistical Analysis

We evaluated the performance of ICD alone, RB-NLP (±ICD), and LLM-NLP (±ICD) for identifying EoE cases against the manually annotated reference standard. Model performance was assessed using the following binary classification metrics: accuracy, precision (PPV), recall (sensitivity), specificity, NPV, F1 score (harmonic mean between recall and precision), and Cohen kappa. Numeric value extraction (eg, eosinophil counts) was assessed using linear correlation (r^2^). To estimate 95% CIs, we applied nonparametric bootstrap resampling (1000 replicates). Statistical significance was determined based on whether the bootstrap-derived 95% CI for the difference compared to the referent excluded zero. McNemar chi-square test was used to compare paired proportions, while chi-square tests evaluated the agreement across case identification methods. For the LLM-NLP approach, time and cost of implementation were estimated. All statistical analyses were performed using Stata (version 18.0; StataCorp) and Python (version 3.12.3; Python Software Foundation), with the latter used for NLP programming. This study was approved by the University of Pennsylvania Institutional Review Board.

## Results

### Development and Validation Cohort Demographics

A random sample of 300 patients was divided into 200 for the development and 100 for the validation cohorts. The median age at biopsy was 52.0 years (interquartile range [IQR]: 41.0–64.0), with younger patients in the validation cohort (49.5 years) compared to the development cohort (53.5 years) (Table 1). Overall, 54% of the cohort was female, with a higher proportion in the development cohort (58%) than in the validation cohort (46%). The majority of patients identified as White (72%), followed by Black (22%), with racial distribution differing between cohorts (White: 66% vs 85%; Black: 28.5% vs 8% in development vs validation, respectively). A total of 92 patients (30.7%) met the manually annotated reference standard for EoE diagnosis, with a higher proportion in the validation cohort (36%) than in the development cohort (28%).

**Table 1:** Demographics and Clinical Characteristics of the Development and Validation Cohorts

Variable	Total cohort (N = 300)	Development cohort (N = 200)	Validation cohort (N = 100)
Demographics, n (%)			
Age at biopsy	52.0 (41.0–64.0)	53.5 (42.0–64.0)	49.5 (39.0–63.5)
Female	161 (54)	115 (58)	46 (46)
Male	139 (46)	85 (43)	54 (54)
Race, n (%)			
Asian	4 (1)	2 (1.0)	2 (2)
Black or African American	65 (22)	57 (29)	8 (8)
Multiracial	1 (0.3)	1 (0.5)	0 (0)
Patient declined	1 (0.3)	1 (0.5)	0 (0)
Some other race	7 (2)	3 (1.5)	4 (4)
Unknown	6 (2)	5 (2.5)	1 (1)
White	216 (72)	131 (66)	85 (85)
Ethnicity, n (%)			
Hispanic Latino	8 (3)	5 (3)	3 (3)
Not Hispanic or Latino	292 (97)	195 (98)	97 (97)
Histology, n (%)			
Positive eosinophils	220 (73)	146 (73)	74 (74)
Esophageal localization of eosinophils	162 (54)	108 (54)	54 (54)
Eosinophil counts increased (≥15 eos/hpf)	103 (34)	59 (30)	44 (44)
Eosinophil count	20.0 (6.0–50.0)	14.0 (5.5–48.0)	24.0 (8.0–59.0)
Symptoms and history, n (%)			
Dysphagia	154 (51)	108 (54)	46 (46)
Food impaction	57 (19)	45 (23)	12 (12)
Reflux symptoms	182 (61)	131 (66)	51 (51)
Documented past EoE history	56 (19)	32 (16)	24 (24)
Reference standard EoE diagnosis	92 (31)	56 (28)	36 (36)
EoE diagnostic code	81 (27)	49 (25)	32 (32)

Histology and symptom features extracted via manual annotation.

### ICD-Only Performance

Among the validation cohort, the ICD-only method yielded precision (PPV) of 0.97 (0.91–1.00) and accuracy of 0.94 (0.89–1.00), with moderate recall (0.86 [0.75–0.97]) and kappa of 0.87 (0.76–0.97), consistent with an F1 score of 0.91 (0.84–1.00) (Table 2).

**Table 2:** LLM-Based NLP Development and Validation Performance Metrics, Reported as Point Estimates With 95% Confidence Intervals (CI)

Method	Precision (PPV)	Recall (sensitivity)	Specificity	NPV	Accuracy	Kappa	F1	ΔF1 Bootstrap
Development set								
ICD alone	0.86 [0.76–0.96]	0.75 [0.64–0.86]	0.95 [0.92–0.99]	0.91 [0.86–0.95]	0.90 [0.85–0.94]	0.73 [0.62–0.84]	0.80 [0.71–0.89]	–
LLM-features diagnosis	0.85 [0.75–0.94]	0.88 [0.79–0.96]	0.94 [0.90–0.98]	0.95 [0.92–0.99]	0.92 [0.88–0.96]	0.80 [0.71–0.90]	0.86 [0.79–0.93]	–
LLM-assigned diagnosis	0.92 [0.84–1.0]	0.80 [0.70–0.91]	0.97 [0.95–1.0]	0.93 [0.89–0.97]	0.93 [0.89–0.96]	0.81 [0.71–0.90]	0.86 [0.78–0.93]	–
ICD + LLM diagnosis	0.85 [0.76–0.94]	0.91 [0.84–0.99]	0.94 [0.90–0.98]	0.96 [0.93–1.0]	0.93 [0.90–0.97]	0.83 [0.74–0.92]	0.88 [0.81–0.95]	–
Validation set								
ICD alone	0.97 [0.91–1.0]	0.86 [0.75–0.97]	0.98 [0.95–1.0]	0.93 [0.86–1.0]	0.94 [0.89–1.0]	0.87 [0.76–0.97]	0.91 [0.84–1.0]	Referent
LLM-derived features	0.93 [0.85–1.0]	0.81 [0.68–0.94]	0.97 [0.91–1.0]	0.90 [0.83–0.97]	0.91 [0.85–0.97]	0.79 [0.67–0.92]	0.87 [0.77–0.96]	−0.05 [−0.13 to 0.04]
LLM-assigned diagnosis	1.0 [1.0–1.0]	0.75 [0.61–0.89]	1.0 [1.0–1.0]	0.87 [0.80–0.95]	0.91 [0.85–1.0]	0.79 [0.66–0.92]	0.86 [0.77–0.95]	−0.05 [−0.16 to 0.05]
ICD + LLM diagnosis	0.97 [0.91–1.0]	0.92 [0.83–1.0]	0.98 [0.95–1.0]	0.95 [0.90–1.0]	0.96 [0.92–1.0]	0.91 [0.83–1.0]	0.94 [0.89–1.0]	0.03 [−0.01 to 0.07]

In the validation set, the combined ICD + LLM approach demonstrated a numeric improvement in F1 score compared with ICD alone (*P* = .20). Sensitivity was also numerically higher for ICD + LLM vs ICD alone (*P* = .15).

### RB-NLP Performance

The RB-NLP approach achieved balanced precision (PPV) and recall (sensitivity) (both 0.75 [0.61–0.89]), with an accuracy of 0.82 (0.75–0.90) and kappa of 0.61 (0.45–0.77) (Supplementary Table 2). The combined RB + ICD method demonstrated the highest recall (sensitivity) (0.89 [0.79–0.99]) and strong overall performance, with an F1 score of 0.83 (0.73–0.93), accuracy of 0.87 (0.80–0.94), and Cohen kappa of 0.73 (0.59–0.86).

Regarding the performance metrics for individual clinical and histologic variables, the validation dataset revealed that positive eosinophils, eosinophil enumeration, dysphagia, and reflux symptoms were extracted with high precision (PPV >0.85) and F1 scores (all >0.75) (Supplementary Figure 2). Variables such as food impaction and descriptive increases in eosinophils showed lower precision, highlighting variability in extraction performance by concept type. Regarding numeric extraction of peak eosinophil count, the RB-NLP system showed moderate correlation in the validation cohort (R^2^ = 0.48) (Supplementary Figure 3).

### LLM-NLP Performance

In the validation cohort, the combined LLM + ICD method showed the highest overall performance, consistent with the development cohort (Table 2). It achieved a recall of 0.92 (0.83–1.00), precision of 0.97 (0.91–1.00), and accuracy of 0.96 (0.92–1.00), with a Cohen kappa of 0.91 (0.83–1.00) and F1 score of 0.94 (0.89–1.00). Notably, the LLM-assigned diagnosis achieved perfect precision and specificity (1.00), though at the cost of lower recall (0.75 [0.61–0.89]). When compared to the ICD-only referent, both LLM-derived features and LLM-assigned diagnosis had slightly lower F1 scores (ΔF1: −0.05 [95% CI: −0.13 to 0.04] and −0.05 [95% CI: −0.16 to 0.05], respectively), whereas the combined LLM + ICD method demonstrated a modest but favorable improvement (ΔF1: +0.03 [95% CI: −0.01 to 0.07]). Comparing NLP approaches, the LLM + ICD method showed an 11% higher F1 score than RB + ICD (difference: 0.11 [95% CI: 0.04–0.19]; *P* = .004).

To validate the real-world performance of the LLM-NLP in the absence of prelabeled data, we first allowed the model to assign EoE diagnoses on a cohort of 93 patients (7 removed due to inadequate clinical notes) and subsequently verified its predictions against a manual review (Supplementary Methods). The combination of ICD codes and LLM diagnosis achieved the highest recall at 0.93 (0.84–1.00) and F1 score of 0.92 (0.84–0.99), suggesting feasibility of an LLM-first labeling strategy (Supplementary Tables 3 and 4).

**Figure 2:**
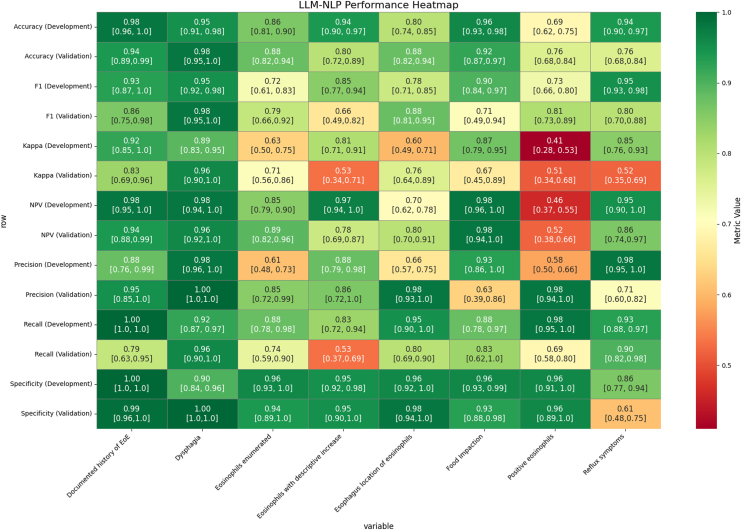
Heatmap of LLM-based NLP development and validation performance metrics for individual variables, point estimates with 95% confidence intervals. Metric values are color-coded from green (high performance) to red (low performance).

LLM-NLP extraction of individual variables displayed strong performance within the development and validation datasets (Figure 2). Only food impaction had a PPV <0.80, while most F1 scores were >0.70, suggesting a balanced trade-off between precision and recall. Regarding numeric extraction of peak eosinophil count, the LLM-NLP system showed moderate correlation (R^2^ = 0.47) (Supplementary Figure 3).

### Comparison of Case Identification Strategies

**Figure 3:**
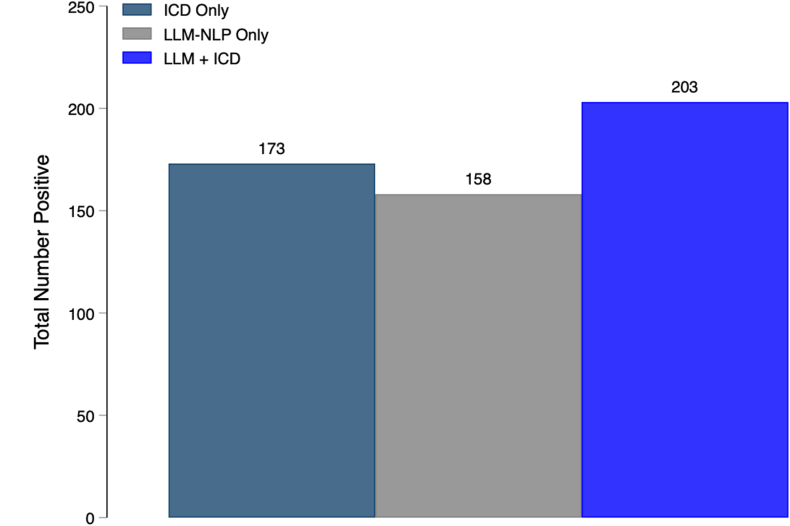
Total number of EoE patients identified by ICD alone, LLM-NLP, and LLM + ICD. LLM + ICD identified more cases than ICD alone (*P* < .001) and LLM-NLP alone (*P* < .001), while there was no significant difference between ICD alone and LLM-NLP alone (*P* = .08).

Because the LLM-NLP strategies demonstrated high performance metrics and validity for an LLM-first labeling strategy, we applied these methods to the remaining unannotated cohort (n = 580) to simulate a larger real-world data set (Supplementary Methods). The LLM + ICD approach identified the highest number (n = 203) of EoE cases, significantly more than either ICD-only (n = 173; *P* < .001) or LLM-NLP-only (n = 158; *P* < .001) (Figure 3). Of the 203 patients identified by the LLM + ICD method, 85% (n = 173) had an ICD code, while 15% (n = 30) were missed by ICD alone. Difference between identification strategies included LLM + ICD cases being younger (median age, 31.0 years [IQR: 26.0–38.0]; *P* = .01), with LLM-NLP only having more dysphagia (87%; *P* < .001) and food impactions (40%; *P* < .001) (Table 3).

**Table 3:** Clinical Characteristics Based on Identification Method

	ICD only (N = 45)	LLM-NLP only (N = 30)	LLM + ICD (N = 128)	No diagnosis (N = 376)	*P*
Demographics, n (%)					
Age at biopsy, median (IQR)	34.0 (27.0–46.0)	36.5 (30.0–49.0)	31.0 (26.0–38.0)	43.5 (29.0–60.0)	.01
Female	17 (38%)	11 (37%)	44 (34%)	201 (54%)	.90
Male	28 (62%)	19 (63%)	84 (66%)	175 (47%)	
Race, n (%)					
Asian	1 (2%)	0 (0%)	8 (6%)	18 (5%)	.50
Black or African American	3 (7%)	5 (17%)	10 (8%)	41 (11%)	
Multi	0 (0%)	0 (0%)	1 (1%)	4 (1%)	
Patient declined	0 (0%)	0 (0%)	0 (0%)	1 (0.3%)	
Other race	0 (0%)	0 (0%)	2 (2%)	14 (4%)	
Unknown	0 (0%)	0 (0%)	0 (0%)	19 (5%)	
White	41 (91%)	25 (83%)	107 (84%)	277 (74%)	
Ethnicity, n (%)					
Hispanic Latino	0 (0%)	0 (0%)	1 (1%)	16 (4%)	.10
Not Hispanic or Latino	43 (96%)	30 (100%)	127 (99%)	357 (95%)	
Histology, n (%)					
Positive eosinophils	34 (76%)	30 (100%)	119 (93%)	138 (37%)	<.001
Eosinophils located in esophagus	34 (76%)	30 (100%)	119 (93%)	91 (24%)	<.001
Eosinophil counts increased (≥15 eos/hpf)	26 (58%)	29 (97%)	103 (81%)	19 (5%)	<.001
Eosinophil count	20.0 (10.0–48.5)	25.0 (20.0–32.0)	30.0 (17.0–70.0)	5.0 (2.0–10.0)	.30
Symptoms and history, n (%)					
Dysphagia	15 (33%)	26 (87%)	102 (80%)	132 (35%)	<.001
Food impaction	8 (18%)	12 (40%)	49 (38%)	29 (8%)	.03
Reflux symptoms	19 (42%)	20 (67%)	78 (61%)	236 (63%)	.05
Documented past EoE history	1 (2%)	5 (17%)	87 (68%)	0 (0%)	<.001
EoE diagnostic code	45 (100%)	0 (0%)	128 (100%)	0 (0%)	<.001

Histology and symptom features are LLM-NLP assigned. Statistical testing excludes the “no diagnosis” column.

### LLM-NLP Time and Cost Estimations

The initial pipeline development required approximately 40 hours for EHR data query and 40–60 hours of programmer time, including prompt engineering (15–20 hours), infrastructure setup (10–15 hours), batch processing implementation (10–15 hours), and evaluation framework development (10–15 hours). Iterative prompt refinement added 20 hours, for a total of 60–80 hours for pipeline development. Processing 50 clinical records with detailed clinical and biopsy reports (1866.0 tokens [IQR: 1305.5–2549.3] per note) consumed approximately $25–$40 in application programming interface costs using GPT-4, translating to roughly $0.50–$0.80 per patient record. For a cohort of 1000 patients, total computational costs would approximate $500–$800. The automated pipeline achieved a processing rate of 10–12 seconds per record (estimated 2–4 hours for 1000 patients), compared to manual chart review by a trained clinician requiring 15–20 minutes per record (250–333 hours per 1000 patients), representing a more rapid approach.

## Discussion

Accurate case identification is critical for limiting bias and enabling high-quality research studies with real-world data.^17^ Many studies on EoE have relied on diagnostic code algorithms, yet this approach can misclassify or fail to capture true cases. Herein, we demonstrate an internally validated and accurate approach using LLM-based NLP techniques that integrate clinical and histologic information, achieving high precision (PPV) that limits false positives. When combined with a single ICD code, the LLM-NLP approach has additional gains in recall (sensitivity), reducing the false negatives with minimal sacrifice to precision. Additionally, the LLM-NLP allows simultaneous extraction of individual variables with a low false-positive rate. Furthermore, when applied to a separate, larger sample, the LLM + ICD pipeline identified additional EoE cases with differences in baseline symptoms and characteristics by identification strategy, suggesting systematic variation between approaches.

Despite the importance of accurate case identification, validated algorithms for EoE remain limited and with notable shortcomings. Both adult and pediatric studies have reported high specificity and low sensitivity, indicating that more than half of cases were missed when relying only on the ICD code.^6^^,^^8^ The aforementioned adult study found differences in clinical characteristics between true positive and false negatives, suggesting potential for selection bias with ICD code reliance. To address challenges with precision, some have proposed more restrictive algorithms, requiring multiple ICD or procedure codes.^7^ While this approach increases precision (PPV), there is a risk of excluding true cases labeled with only a single code, thereby lowering sample size and potentially selecting for patients with more persistent symptoms. The current study revealed that a combination LLM + ICD method reduced false negatives and relied only on a single ICD code without the trade-off of overly restrictive criteria. Moreover, the demographic and clinical differences between identification methods in our study represent a central finding that could have implications for observational research. The LLM-NLP approach identified individuals near the upper limit of the peak incidence age range, which may be related to clinicians more frequently considering alternative diagnoses instead of EoE in older patients. Furthermore, the higher frequency of dysphagia and food impactions in this group may suggest phenotypic variation, whereas individuals identified only by ICD code appeared more often asymptomatic and in histologic remission. These findings warrant further investigation but highlight the potential for selection bias depending on the case ascertainment method, as well as the potential added value of LLM-NLP in improving case identification.

Interest in applying LLMs in gastroenterology is growing.^18^^,^^19^ While RB-NLP systems have been widely used for various task,^20^ their broader adoption has been limited by the programming and linguistic expertise required for implementation. To date, relatively few studies have leveraged LLMs specifically for disease case identification, and even fewer have focused on EoE. One study showed mixed accuracy of LLM responses to patient-oriented questions about EoE.^21^ Studies in other gastrointestinal conditions have demonstrated that LLM-based NLP pipelines can accurately identify conditions such as gastrointestinal bleeding using nursing notes and laboratory data, demonstrating low false-positive and false-negative rates.^22^ Additional work has shown LLMs capable of extracting histopathologic features from colorectal specimens with high precision and recall.^23^ Others have evaluated LLM-based approaches for identifying features of cirrhosis and hepatocellular carcinoma, with authors proposing that LLM-NLP could serve as a sufficient reference standard in lieu of manual annotation.^24^^,^^25^ Building on this prior work, our study demonstrates accurate case identification of EoE using LLM-NLP. We observed comparable performance of an LLM-first approach, supporting its potential as a scalable alternative to traditional manual review. Beyond case identification, our LLM-NLP pipeline also extracted individual diagnostic features with high precision, further demonstrating its potential to enhance or replace manual extraction. Another key innovation of our study is the integration of both clinical and pathology text to assign diagnosis for a clinicopathologic disease. To our knowledge, this represents one of the first applications of LLMs to integrate these text data types and supports the use of LLMs for accurate case ascertainment from real-world data, with or without traditional code-based methods.

Beyond the test characteristics of the NLP approaches, several observations and potential limitations warrant discussion. First, ICD coding for EoE demonstrated higher performance in our cohort than previously reported. When baseline precision is high, as observed here, improvements in recall may yield only modest changes in performance despite identifying additional true cases. This likely reflects institutional expertise and cohort construction, as individuals with ICD codes lacking confirmatory pathology may have been excluded, reducing false positives and potentially limiting generalizability to cohorts constructed differently. However, this does not diminish the utility of the LLM-NLP approach, which reduced false positives while identifying additional cases missed by ICD alone. Prior studies support the robustness of LLM-based approaches across settings where ICD performance varies, but more testing is required to address this with our approch.^26^

Second, extraction of individual variables showed differences between methods, where LLM-NLP generally outperformed RB-NLP, though RB-NLP performed well for highly structured tasks. This aligns with prior work showing superior performance of LLM-NLP for symptom extraction,^27^ though others have proposed advantages to a hybrid approach.^22^ More testing is necessary to clarify this matter. Third, our analysis was restricted to the prebiopsy clinic notes to preserve temporality and due to computational and token constraints (ie, limits on the amount of text that can be processed), limiting review of the full patient record (eg, general medicine or allergy/immunology clinics). Inclusion of all documents could have enhanced performance, and external records might have strengthened generalizability. At present, broader application of LLM-NLP approaches face practical limitations, but studies are underway to understand multisite applicability. Fourth, we used an off-the-shelf LLM (OpenAI models), which simplified implementation, but alternative models may offer better performance, cost, or explainability. Lastly, the reference standard was annotated by a single reviewer, which may introduce misclassification bias; however, LLM-based case ascertainment is an evolving area, and future studies will examine multireviewer annotation and formal interrater agreement to strengthen validation. Ongoing efforts to address these limitations will advance the use of LLMs for real-world data extraction.

Several strengths of this study are worth emphasizing. We introduce an accurate approach using LLM-based NLP that has potential to enhance or replace code-based methods for cohort identification while enabling the simultaneous extraction of the individual diagnostic variables. Application of NLP methods within secure computational environments, coupled with structured prompt development, ensured robustness and consistency of data extraction. Additionally, the human-in-the-loop approach allows for human oversight with ongoing refinement, supporting adaptable, goal-specific applications.^16^^,^^28^ We acknowledge demographic imbalance between the development and validation cohorts; however, this may be considered a strength since clinical features often vary across populations and sites. Additionally, while the F1 increase for LLM + ICD over ICD-only was modest, the LLM-NLP method captured 30 additional EoE cases (15% increase vs ICD alone) in the larger sample, demonstrating that small metric gains in validation can yield meaningful improvements in real-world case identification. The impact of such gains is likely to increase as these methods scale to larger datasets. Finally, we highlight the cost and time required to implement LLM-NLP pipelines, an important yet underexplored consideration in the current literature. Some studies have demonstrated cost benefits, particularly with multiple queries per document, similar to our approach.^13^^,^^29^ While further research is needed to define cost-to-benefit tradeoffs, our approach suggests that, for large-scale studies, months of manual annotation work would be reduced to days of automated processing, with the primary time investment shifted to quality assurance and validation rather than primary data extraction.

## Conclusion

We developed and validated an LLM-based NLP approach, revealing that the LLM-NLP pipeline has high performance metrics for identifying EoE patients and extracting diagnostic variables. This approach offers the capability for consistent, large-scale data extraction, potentially reducing both manual effort and associated costs. When applied to a larger cohort, the LLM-NLP method identified additional cases, revealing clinical differences based on identification strategy. Combining LLM-NLP with a single diagnostic code offers a scalable and accurate alternative to traditional code-based algorithms while enabling simultaneous extraction of individual diagnostic variables. These tools have the potential to enhance real-world data pipelines, improve data quality, and advance research in EoE and other clinicopathologic conditions. Further work is needed to evaluate scalability and generalizability across diverse data sources, diseases, and health-care settings.

## Supplementary Materials

Supplementary Methods

Extended PDF
