# Supplementary material for: Development and Validation of a Large Language Model Case Identification Strategy for Eosinophilic Esophagitis
Source: Gastro Hep Adv. 2026 Apr 16;5(7):100971. doi: 10.1016/j.gastha.2026.100971 (PMC13207557; doi:10.1016/j.gastha.2026.100971)

Supplemental Tables and Figures

Supplemental Table 1. Definitions of the 10 variables extracted from the relevant clinical and histopathologic text.

**Variable (name)**	**Definition**
Positive eosinophils (“eos_present”)	Indicates whether eosinophils are positively mentioned in any part of the biopsy report; answer “No” if explicitly stated as absent throughout (“no eosinophils”) or no mention of eosinophils.
Eosinophils located in the esophagus (“eos_location___esoph”)	Specifies if eosinophils are detected in esophageal tissue; answer “No” if they are absent, unclear, or only described in other anatomic locations.
Eosinophils enumerated (“eos_count”)	Indicates whether a specific numeric eosinophil count per high-power field (eos/hpf) is reported in the biopsy report.
Peak eosinophil count (“esoph_eos_num”)	The highest reported eosinophil count (per high-power field) in any esophageal biopsy section. If multiple values are present, select the maximum; if no numeric value is given for the esophagus, enter “0”.
Descriptive increase of eosinophils (“esoph_eos_num_desc_increased”)	Indicates whether there is descriptive language in the esophageal biopsy suggesting increased eosinophils, including terms like “increased”, “numerous”, or “eosinophilic infiltration”.
Dysphagia (“dysphagia”)	Presence of dysphagia symptoms (trouble swallowing, food sticking, etc.) in the clinical report. Must be non-negated. Historic or intermittent symptoms qualify.
Food impation (“food_impact”)	Indicates whether there is mention of food impaction (food getting stuck or obstructing swallowing), which is more severe than dysphagia alone. Requires explicit or synonymous terminology.
Atypical esophageal symptoms (“reflux”)	Presence of reflux, heartburn, regurgitation or related diagnosis noted in the clinical report.
Documented past EoE history (“pmh_eoe”)	Whether the patient has a prior diagnosis or history of eosinophilic esophagitis (EoE) listed in the clinical report. Mention must refer to established history, not just differential.
EoE diagnosis (“eoe_dx”)	Indicates if the patient meets criteria for a diagnosis of EoE based on a combination of biopsy and clinical report findings (i.e., increased eosinophils in the esophagus and relevant symptoms, or documented history of EoE).

Supplemental Figure 1. Human-in-the-loop process for development of large language model (LLM) pipeline to extract clinical variables from unstructured text.

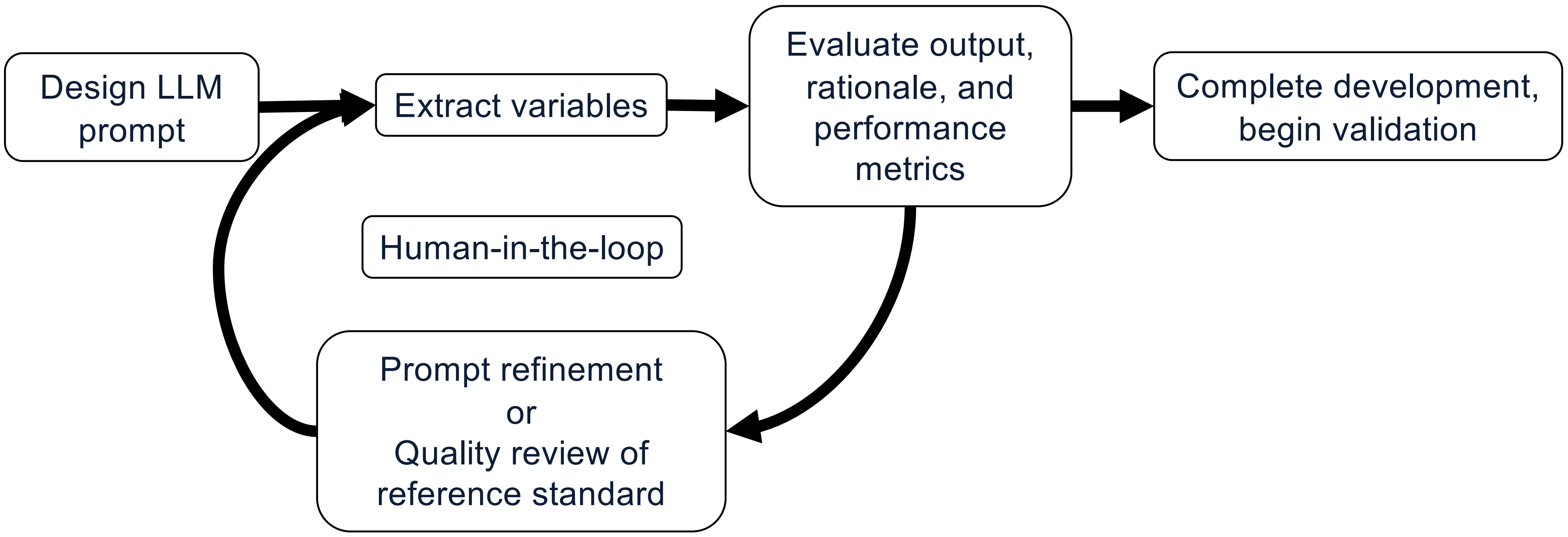

Supplemental Table 2. Rules-based NLP development and validation performance metrics, point estimates with 95% confidence intervals.

Method	Precision (PPV)	Recall (Sensitivity)	Specificity	NPV	Accuracy	Kappa	F1
**Development Set**							
**ICD alone**	0.86 [0.76,0.96]	0.75 [0.64,0.86]	0.95 [0.92,0.99]	0.91 [0.86,0.95]	0.90 [0.85,0.94]	0.73 [0.62,0.84]	0.80 [0.71,0.89]
**RB-NLP diagnosis**	0.72 [0.61,0.82]	0.86 [0.77,0.95]	0.87 [0.81,0.92]	0.94 [0.90,0.98]	0.87 [0.82,0.91]	0.68 [0.57,0.80]	0.78 [0.69,0.87]
**ICD+RB diagnosis**	0.71 [0.60,0.81]	0.95 [0.89,1.0]	0.85 [0.79,0.91]	0.98 [0.95,1.0]	0.88 [0.83,0.92]	0.72 [0.62,0.82]	0.81 [0.73,0.89]
**Validation Set**							
**ICD alone**	0.97 [0.91,1.0]	0.86 [0.75,0.97]	0.98 [0.95,1.0]	0.93 [0.86,1.0]	0.94 [0.89,1.0]	0.87 [0.76,0.97]	0.91 [0.84,1.0]
**RB-NLP diagnosis**	0.75 [0.61,0.89]	0.75 [0.61,0.89]	0.86 [0.77,0.95]	0.86 [0.77,0.95]	0.82 [0.75,0.90]	0.61 [0.45,0.77]	0.75 [0.62,0.88]
**ICD+RB diagnosis**	0.78 [0.65,0.91]	0.89 [0.79,0.99]	0.86 [0.77,0.95]	0.93 [0.87,1.0]	0.87 [0.80,0.94]	0.73 [0.59,0.86]	0.83 [0.73,0.93]

Supplemental Figure 2. Rules-based NLP development and validation performance metrics for individual variables, point estimates with 95% confidence intervals.

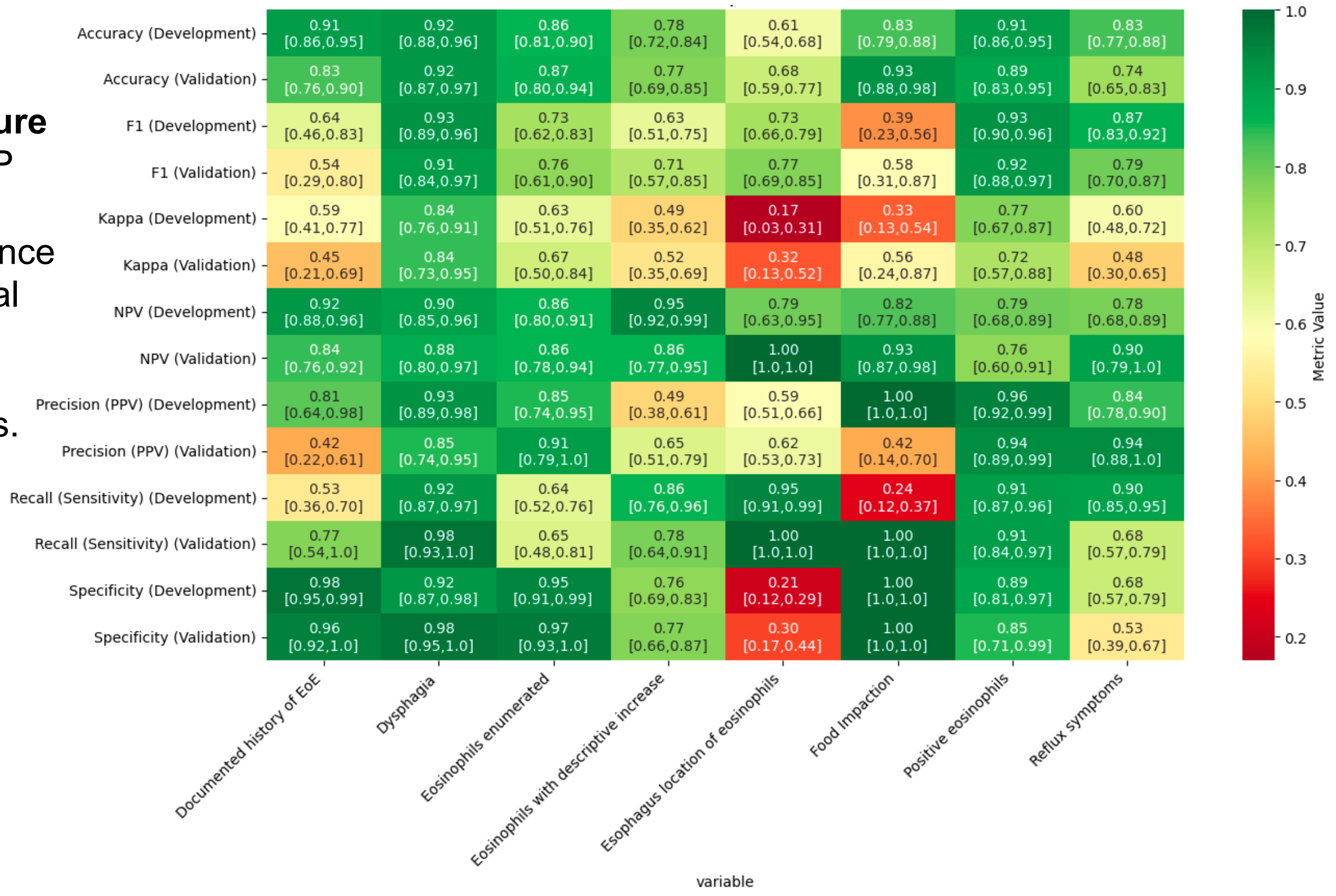

Supplemental Table 3. Demographics and clinical characteristics of the LLM-first cohort. Histology and symptom features are LLM-NLP assigned.

	LLM-first validation (N=93)
**Demographics, n(%)**	
Age at biopsy	46.5 (35.0-62.0)
Female	60 (60)
Male	40 (40)
**Race, n(%)**	
Asian	3 (3)
Black or African American	11 (11)
Multiracial	1 (1)
Patient Declined	1 (1)
Some Other Race	3 (3)
Unknown	2 (2)
White	79 (79)
**Ethnicity, n(%)**	
Hispanic Latino	2 (2)
Not Hispanic or Latino	97 (97)
**Histology, n(%)**	
Positive eosinophils	47 (47)
Eosinophils located in esophagus	42 (42)
Eosinophil counts increased (≥15 eos/hpf)	31 (31)
Eosinophil count	30.0 (6.0-50.0)
**Symptoms and history, n(%)**	
Dysphagia	50 (50)
Food impaction	18 (18)
Reflux symptoms	59 (59)
Documented past EoE history	15 (15)
Manually annotated EoE diagnosis	29 (29)
EoE diagnostic code	34 (34)

Supplemental Table 4. LLM-NLP-first development and validation performance metrics, point estimates with 95% confidence intervals.

Method	Precision (PPV)	Recall (Sensitivity)	Specificity	NPV	Accuracy	Kappa	F1
ICD	0.90 [0.79,1.0]	0.90 [0.79,1.00]	0.95 [0.90,1.0]	0.95 [0.90,1.0]	0.94 [0.89,0.99]	0.85 [0.73,0.97]	0.90 [0.81,0.98]
LLM-derived features	0.93 [0.83,1.0]	0.90 [0.79,1.0]	0.97 [0.93,1.0]	0.95 [0.90,1.0]	0.95 [0.90,0.99]	0.87 [0.77,0.98]	0.91 [0.83,0.99]
LLM-assigned diagnosis	1.0 [1.0,1.0]	0.79 [0.65,0.94]	1.0 [1.0,1.0]	0.91 [0.85,0.98]	0.94 [0.89,0.99]	0.84 [0.72,0.96]	0.89 [0.79,0.98]
ICD+LLM diagnosis	0.90 [0.79,1.0]	0.93 [0.84,1.0]	0.95 [0.90,1.0]	0.97 [0.93,1.0]	0.95 [0.90,0.99]	0.91 [0.83,1.0]	0.92 [0.84,0.99]

Supplemental Figure 3. Scatter plots comparing eosinophil counts extracted by the rules-based NLP pipeline to manually annotated reference-standard counts in esophageal biopsy reports for both (A) RB-NLP validation and (B) LLM-NLP validation. The blue line indicates the best-fit linear regression.

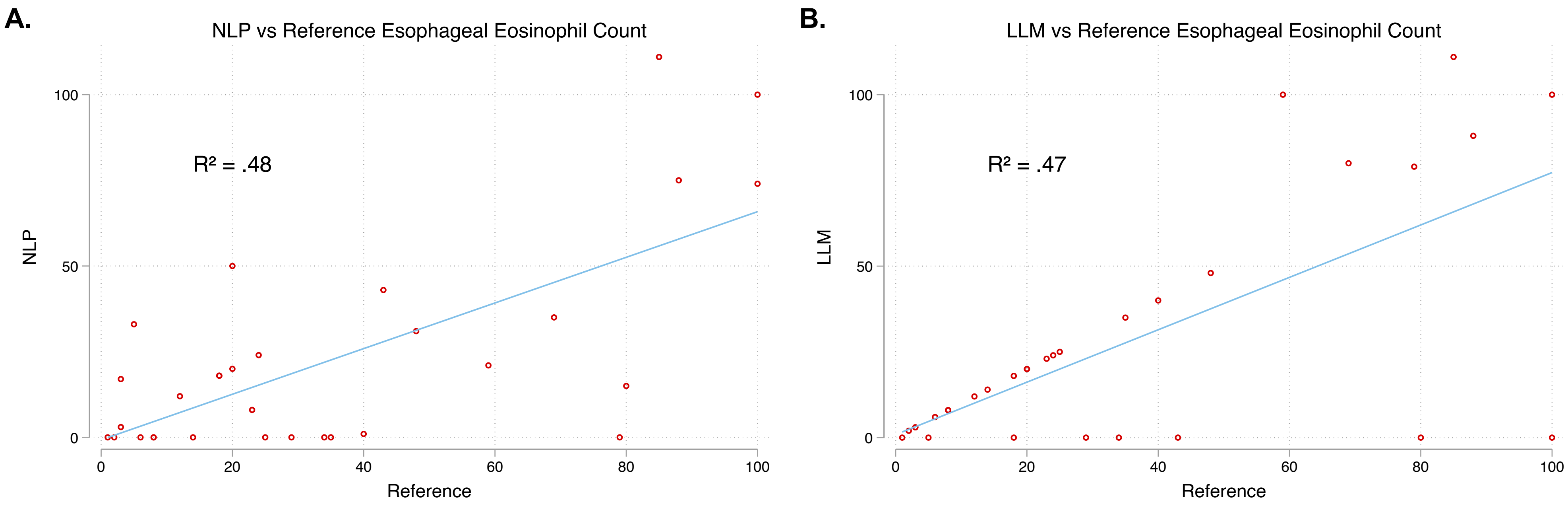

Supplement: Supplementary Tables and Figures [file mmc1.pdf]
