# Supplementary material for: Development and Validation of a Large Language Model Case Identification Strategy for Eosinophilic Esophagitis
Source: Gastro Hep Adv. 2026 Apr 16;5(7):100971. doi: 10.1016/j.gastha.2026.100971 (PMC13207557; doi:10.1016/j.gastha.2026.100971)
Supplement: Supplementary Methods [file mmc2.pdf]

SUPPLMENTAL METHODS

To develop the cohort, we queried Penn Medicine patients with clinical encounters from 2008 through April 2024 with patients initially identified by surgical pathology procedure orders (Figure 1). The Penn Medicine system includes the Hospital of the University of Pennsylvania, Pennsylvania Hospital, Penn Presbyterian Medical Center, and several smaller primarily outpatient facilities. For NLP pipeline development and validation, we included patients with at least one associated clinical note occurring between 1 and 60 days prior to the corresponding biopsy date to ensure extracted clinical information reflected the pre-biopsy context. This filtering narrowed the calendar years of cases between 2015 through April 2024. From a sample of 1,000 patients meeting these criteria, we randomly allocated them to a development cohort (n=200; 56 with EoE) and a validation cohort (n=100; 36 with EoE). This approach allowed for a temporally-aligned, filtered cohort suitable for downstream NLP and analysis. Assuming a PPV of 80%, the sample sizes yielded 95% confidence intervals with margins of error ranging from ±9% to ±11%. Due to token (smaller fundamental units of text) limitations, the first clinic and biopsy notes per patient were analyzed during the NLP tasks. Only observations with complete data were used. Manual chart review (performed by CJK, gastroenterologist) of the deidentified clinic note and biopsy report was used to assign the reference standard for key clinical variables and EoE diagnosis (symptoms + positive histology or history of EoE) in both development and validation cohorts (Supplemental Table 1).

RB-NLP Pipeline

RB-NLP was constructed on top of spaCy with medSpaCy's clinical components enabled. For sentence segmentation, we applied spaCy's sentencizer preceded by a custom boundary-setting component to improve segmentation accuracy around bullet points and section headers. For entity extraction, we curated a set of TargetRule definitions using medSpaCy's TargetMatcher to identify key clinical concepts relevant to EoE (e.g., "trouble swallowing" → dysphagia). These rules produced binary features indicating whether each concept was present anywhere in the document (Supplemental Table 1). Eosinophil counts per high-power field (HPF) were extracted using regular expressions. Because descriptive eosinophil terminology can appear outside structured count statements, we manually evaluated these descriptors and found that phrases indicating increased eosinophils corresponded to a median peak of 35 eos/HPF (IQR 20.5–51.5). Accordingly, these descriptions were operationalized as meeting the ≥15 eos/HPF threshold for EoE. For assertion detection, we incorporated

medSpaCy's ConText component to determine whether extracted entities were negated ("no eosinophils"), historical ("history of EoE"), or modified by intensity or temporality.

Diagnostic decision logic for both the reference standard (human applied) and NLP approaches:

- A patient was classified as EoE if either
    1. a historical diagnosis of EoE was identified in the clinic note (not negated), or
    2. pathology features extracted from the pathology report met EoE histologic criteria (≥15 eos/HPF or descriptive increases mapped to this threshold) AND features extracted from the clinic note met EoE symptom criteria .
- All extracted features were evaluated at the document level; if any qualifying feature appeared in the note and was not negated or historical-only, it contributed to the classification.
- No machine-learning classifier was used; classification relied entirely on deterministic rule-based thresholds.

RB-NLP predictions were compared with the manually annotated reference standard using binary classification metrics. The programming code for the RB-NLP pipeline is available upon request.

LLM-NLP Pipeline

For the LLM portion of the project, we followed TRIPOD-LLM reporting guidelines.(1) During prompt development, we used established clinical criteria and domain knowledge relevant to EoE diagnosis. Utilizing human-in-the-loop iterative refinement process, we aimed to address several failure modes encountered during early testing (Supplemental Figure 1).(2) These included (i) misinterpretation of negated or ambiguous phrases, (ii) difficulty distinguishing historical from current diagnoses, and (iii) inconsistent recognition of eosinophil descriptors that lacked explicit numerical counts (e.g., "increased eosinophils," "numerous eosinophils"). To mitigate these issues, we structured the prompt around established EoE diagnostic criteria and incorporated balanced positive and negative examples for each binary field, including common negations and linguistically ambiguous terms ("rare eosinophils," "negative for eosinophils"). This design was motivated by the need to

ensure consistent interpretation of subtle clinical language and to reduce hallucinated or unsupported extractions.

Source text was segmented by section headers (e.g., BIOPSY REPORT, CLINICAL REPORT) to help the model correctly anchor eosinophil counts and diagnostic statements in their proper clinical context. Numeric eosinophil values were extracted using deterministic rules but verified by the LLM to address transcription-like inconsistencies across notes. We also required the model to provide textual evidence and a brief rationale for each output to promote transparency, allow interrogation of failure cases, and streamline quality assessment.

After each iteration, LLM outputs, rationales, and misclassified examples were reviewed to determine whether errors were attributable to prompt limitations or reference standard issues. Refinement continued until the LLM-assigned diagnosis exceeded the F1-score of ICD alone. A temperature of 0.2 was used throughout development to improve determinism. Once performance stabilized and no further failure modes emerged, model development was concluded.

Additional LLM-NLP Validation in Unannotated Cohorts

Using the remaining unannotated and unseen cases, we evaluated the performance of an LLM-first labeling approach. The LLM-NLP pipeline was applied prior to assigning the reference standard, allowing retrospective validation with knowledge of outputs. This strategy enables assessment of LLM-NLP scalability compared to a manual annotation-first approach. We measured performance metrics for the LLM-assigned diagnosis of EoE on 93 cases (7 removed for inadequate documentation). Further, we applied the pipeline to 580 cases to quantify the number of EoE cases identified with ICD alone, LLM-NLP alone, and LLM-ICD combination. Demographics and LLM-NLP derived clinical features were compared by case identification strategies.

Performance Metric Definitions (Lay Explanation)

To aid interpretation of model performance, we provide simplified explanations of the evaluation metrics reported in this study:

- Accuracy: The proportion of all predictions the model gets right.

- Example: How often the model correctly identifies whether a patient does or does not have EoE.
- Precision (Positive Predictive Value, PPV): When the model predicts that a patient has EoE, how often that prediction is correct.
  - Example: “Of all the patients the model labeled as EoE, how many truly had the condition?”
- Recall (Sensitivity): How well the model finds true cases.
  - Example: “Of all patients who truly had EoE, how many did the model successfully identify?”
- Specificity: How well the model avoids false alarms.
  - Example: “Of all patients who did NOT have EoE, how many did the model correctly identify as not having it?”
- F1 Score: A combined measure of precision and recall, balancing how well the model avoids false positives and how well it captures true positives.
  - Example: A single number that summarizes both “how many true cases the model finds” and “how often those identifications are correct.”

Structured information extraction prompt for LLM-NLP pipeline to identify key EoE variables.

Finalized Prompt
You are a specialized medical information extraction system with expertise in gastroenterology and pathology, with a focus on eosinophilic gastrointestinal diseases (EGIDs), including eosinophilic esophagitis (EoE)<br>TASK: Extract specific structured information from the following medical note about eosinophils, EGID diagnosis, and related symptoms. Read the ENTIRE note thoroughly before answering.<br>CLINICAL AND BIOPSY REPORT:<br>{text}<br>EXTRACTION GUIDELINES:<br>1. Eosinophils Present (eos_present):<br>- Only evaluate the text in the BIOPSY REPORT. Ignore all text that appears before this section.<br>- Answer "Yes" if ANY of these conditions are met:<br>* There is a positive mention of the terms: "eosinophil(s)", "intraepithelial eosinophils", "eosinophilic", or "eosinophilia", without a negation.<br>* Positive descriptive qualifiers include "few", "rare", "scattered", "present", "increased", or "prominent" eosinophils.<br>- Answer "No" ONLY if:<br>* The report explicitly states absence of eosinophils using terms like: "no eosinophils", "no evidence of", "absent", "not seen", "negative for eosinophils, “no increase in intraepithelial eosinophils”, or similar phrases indicating lack of histologic increase.<br>- IMPORTANT:<br>* If eosinophils are mentioned with any non-negated descriptor, even if minimal (e.g., “rare eosinophils present”), answer “Yes”.<br>* Be cautious with negation terms near “eosinophil” mentions. A negated context (e.g., “no eosinophils seen”) should be interpreted as “No”.<br>* Mentions of blood eosinophil levels (e.g., “eosinophil count in blood”, “peripheral eosinophilia”) from a CBC are not considered evidence of eosinophils in the biopsy.<br>2. Eosinophils in Esophagus (eos_location___esoph):<br>- Only evaluate the text in the BIOPSY REPORT. Ignore all content before this section.<br>- Answer "Yes" if ANY of these conditions are met:<br>* Eosinophils are described in a section clearly labeled or referring to esophageal tissue, including (but not limited to) the following headers or phrases: "Esophagus, biopsy:", "Esophagus:", "Mid esophagus", "Distal esophagus", "Proximal esophagus", "Gastroesophageal", "Esophagogastric", or "Esophageal-gastric junction"<br>* There is a mention of eosinophil-related findings (e.g., "increased eosinophils", "eosinophilic infiltrate", "rare eosinophils", "eosinophil count", or "increased intraepithelial eosinophils") within or immediately following an esophageal section, with no intervening section header indicating a different anatomical site.<br>- Answer "No" if:<br>* Eosinophils are only described in non-esophageal locations, such as the stomach, duodenum, colon, ileum, or any other labeled section not referring to the esophagus.<br>* Eosinophils are explicitly stated as absent, not seen, no evidence, or negative in the esophageal section.

* The anatomical location where eosinophils are observed is unclear or cannot be confidently attributed to the esophagus.
* There is no mention of eosinophils in association with esophageal tissue.
- IMPORTANT:
* Use section headers as anchors to assign findings to a specific location.
* When eosinophils are described after an "Esophagus" header, assume they apply to that location unless a new header appears.
* Mentions of eosinophils in blood or systemic references (e.g., “peripheral eosinophilia”) should be ignored.

3. Eosinophil Count Provided (eos_count):
- Only evaluate the text in the BIOPSY REPORT. Ignore all text that appears before this section.
- Answer "Yes" if ANY of these conditions are met in the biopsy report:
* ANY specific number or range of eosinophils is mentioned (e.g., “15 eosinophils per high power field”, “15 eos/HPF”, “15 eos/hpf”, “15/hpf”, “20–30 eosinophils per HPF”)
* A numeric threshold is provided (e.g., "eosinophils >15/HPF", "less than 5 eos/hpf")
* A count of zero is numerically specified (e.g., “0 eosinophils/HPF” or “no eosinophils (0/HPF)”
* The eosinophil count appears in a section labeled as or clearly referring to the esophagus (e.g., "Esophagus", "Mid esophagus", etc.).
- Answer "No" if:
* Only qualitative descriptions are given (e.g., "few eosinophils", "many", "prominent", "increased", "rare"), without a numeric value.
* No specific numeric value related to eosinophils appears anywhere in the biopsy report section.

4. Esophageal Eosinophil Number (esoph_eos_num):
- Only evaluate the text in the BIOPSY REPORT. Ignore all text that appears before this section.
- In the biopsy report, Identify all numeric eosinophil counts per HPF reported for esophageal locations, and extract the single highest value.
* For a single value (e.g., "40 eos/HPF"), report just "40"
* For a range (e.g., "0-1 eos/HPF"), report the "1"
* For a threshold (e.g., ">15 eos/HPF"), report "15"
* For multiple counts from different locations in the esophagus, report the HIGHEST value
- Report "0" if no specific eosinophil count is provided for the esophagus
- Examples of correct extraction:
* From "20 eosinophils per high-power field" ? "20"
* From "eosinophil counts ranging from 5-30/HPF" ? "30"
* From "proximal: 10 eos/HPF, distal: 45 eos/HPF" ? "45"

5. Increased Eosinophil Description (esoph_eos_num_desc_increased):
- Only evaluate the text in the BIOPSY REPORT. Ignore all text that appears before this section.
- Answer "Yes" if ANY of these conditions are met:
*The biopsy report includes descriptive language indicating increased or elevated eosinophils in the esophagus, such as: "increased eosinophils", "elevated eosinophils", "numerous eosinophils", "many eosinophils", "marked eosinophilia", "dense eosinophilic infiltrate", "eosinophil-rich inflammation"
* The pathologist implies increased eosinophilic activity or disease even without using the word "increased", such as: "eosinophilic infiltration", "expansion of eosinophils", "active esophagitis with eosinophils"
* Pathologist interpretation suggesting increased eosinophils even without the explicit term "increased"
* A mention of "active" disease
- Answer "No" if:
* Eosinophils are described as "rare", "few", "occasional", "scant", or "normal"
* Only a numeric count is given without qualitative description
* No qualitative assessment of eosinophil quantity is provided
- NOTE: If both a qualitative description AND a numeric count are given, still answer "Yes" if the description indicates increase

6. Dysphagia (dysphagia):
- Focus on the CLINICAL REPORT
- Answer "Yes" if ANY of these conditions are met:
* The term "dysphagia" appears and is not negated
* Patient reports difficulty swallowing or food getting stuck
* Descriptions of "trouble swallowing", "choking sensation", “food moving slowly”, or "food sticking" in throat/chest
- Answer "No" if:
* Such symptoms are explicitly denied (e.g., "no dysphagia", "denies difficulty swallowing")
* There is no mention of swallowing difficulties
- IMPORTANT: Even historic or intermittent dysphagia should be marked "Yes"

7. Food Impaction (food_impact):
- Focus on the CLINICAL REPORT
- Answer "Yes" if ANY of these conditions are met:
* Explicit mention of "food impaction" or "food bolus obstruction"
* Descriptions of food or bolus getting stuck
* Patient reporting "food or liquids won't go down" or "complete blockage"
* History of endoscopy to remove food impaction
* Terms like "impaction", "obstruction", or "sticking" in relation to food
- Answer "No" if:
* Only mild dysphagia without complete obstruction is reported
* No mention of food getting stuck
* Food-related symptoms are explicitly denied
- NOTE: Food impaction is more severe than dysphagia alone; it involves food getting completely stuck

8. Reflux (reflux):
- Focus on the CLINICAL REPORT
- Answer "Yes" if ANY of these conditions are met:
* Direct mention of reflux symptoms, such as "reflux", "GERD", “burning in chest”, “heartburn”, “regurgitation”, or "gastroesophageal reflux"
* Past medical history of reflux
* Medications with an indication to treat reflux only
- Answer "No" if:
* Reflux is explicitly denied (e.g., "no reflux symptoms", "denies GERD")
* No mention of reflux or GERD anywhere in the note

9. Past Medical History of EoE (pmh_eoe):
- Focus on the CLINICAL REPORT

- Answer "Yes" if ANY of these conditions are met:
  * The clinical report states a past medical history of "eosinophilic esophagitis", "EoE", "esophageal eosinophilia", or "PPI-responsive esophageal eosinophilia".
  * Terms like "known EoE", "established EoE", "history of EoE", "previously diagnosed with EoE", or "#Eosinophilic esophagitis"
  * EoE listed under "Past Medical History" or "PMH" sections
- Answer "No" if:
  * EoE is mentioned only as a differential diagnosis
  * No indication of a pre-existing EoE diagnosis before current encounter

10. EoE Diagnosis (eoe_dx):
- Use information from both the CLINICAL REPORT and the BIOPSY REPORT to determine whether the patient meets criteria for eosinophilic esophagitis (EoE).
- Answer "Yes" if either of the following is true:
  * The clinical report states a past medical history of "eosinophilic esophagitis", "EoE", "esophageal eosinophilia", or "PPI-responsive esophageal eosinophilia".
  * The BIOPSY REPORT has a descriptive increase in eosinophils in the esophagus (e.g., “mildly increased,” “moderately increased,” or “increased”) OR a numerical count ≥15 eosinophils per high power field (eos/hpf) AND the CLINICAL REPORT includes symptoms consistent with EoE, such as dysphagia, food impaction, chest pain, heartburn, or regurgitation.
- If none of the above criteria are met, answer "No".

IMPORTANT CONTEXT:
In pathology reports, the following findings often indicate EoE:
- Basal zone hyperplasia
- Dilated intercellular spaces (spongiosis)
- Subepithelial fibrosis
- Surface layering of eosinophils or eosinophil microabscesses

RESPONSE FORMAT:
For each field, provide your answer AND a brief rationale citing direct evidence from the note:
eos_present: Yes/No
Rationale: [Cite specific text and explain your reasoning]
eos_location___esoph: Yes/No
Rationale: [Cite specific text and explain your reasoning]
eos_count: Yes/No
Rationale: [Cite specific text and explain your reasoning]
esoph_eos_num: [exact number or "0"]
Rationale: [Cite specific text and explain your reasoning]
esoph_eos_num_desc_increased: Yes/No
Rationale: [Cite specific text and explain your reasoning]
dysphagia: Yes/No
Rationale: [Cite specific text and explain your reasoning]
food_impact: Yes/No
Rationale: [Cite specific text and explain your reasoning]
reflux: Yes/No
Rationale: [Cite specific text and explain your reasoning]
pmh_eoe: Yes/No
Rationale: [Cite specific text and explain your reasoning]
eoe_dx: Yes/No
Rationale: [Cite specific text and explain your reasoning]
YOU MUST PROVIDE A DETAILED RATIONALE FOR EACH FIELD, EVEN IF THE ANSWER IS "NO" OR "0".

REFERENCES

1. Gallifant J, Afshar M, Ameen S, et al. The TRIPOD-LLM reporting guideline for studies using large language models. Nat Med 2025;31:60-69.

2. Wang ZJ, Choi D, Xu S, et al. Putting humans in the natural language processing loop: A survey. arXiv preprint arXiv:2103.04044 2021.

The TRIPOD-LLM Statement: A Targeted Guideline For Reporting Large Language Models Use

Supplementary Table 2: Fillable TRIPOD-LLM checklist

Section	Item	Checklist Item	Research Design	LLM Task	Page
Title					
Title	1	Identify the study as developing, fine-tuning, and/or evaluating the performance of an LLM, specifying the task, the target population, and the outcome to be predicted.	All	All	4, 5
Abstract					
Abstract	2	See TRIPOD-LLM for Abstracts	All	All	
Introduction					
Background	3a	Explain the healthcare context / use case (e.g., administrative, diagnostic, therapeutic, clinical workflow) and rationale for developing or evaluating the LLM, including references to existing approaches and models.	All	All	4
	3b	Describe the target population and the intended use of the LLM in the context of the care pathway, including its intended users in current gold standard practices (e.g., healthcare professionals, patients, public, or administrators).	E<br>H	All	4
Objectives	4	Specify the study objectives, including whether the study describes the initial development, fine-tuning, or validation of an LLM (or multiple stages).	All	All	4
Methods					
Data	5a	Describe the sources of data separately for the training, tuning, and/or evaluation datasets and the rationale for using these data (e.g., web corpora, clinical research/trial data, EHR data, or unknown).	All	All	5, Supplemental
	5b	Describe the relevant data points and provide a quantitative and qualitative description of their distribution and other relevant descriptors of the dataset (e.g., source, languages, countries of origin)	All	All	5, Supplemental
	5c	Specifically state the date of the oldest and newest item of text used in the development process (training, fine-tuning, reward modeling) and in the evaluation datasets.	All	All	5, Supplemental
	5d	Describe any data pre-processing and quality checking, including whether this was similar across text corpora, institutions, and relevant socio-demographic groups.	All	All	Supplement
	5e	Describe how missing and imbalanced data were handled and provide reasons for omitting any data.	All	All	Supplement
Analytical Methods	6a	Report the LLM name, version, and last date of training.	All	All	6
	6b	Report details of LLM development process, such as LLM architecture, training, fine-tuning procedures, and alignment	M<br>D	All	NA

		strategy (e.g., reinforcement learning, direct preference optimization, etc.) and alignment goals (e.g., helpfulness, honesty, harmlessness, etc.).			
	6c	Report details of how text was generated using the LLM, including any prompt engineering (including consistency of outputs), and inference settings (e.g., seed, temperature, max token length, penalties), as relevant.	M D E	All	Supplemental
	6d	Specify the initial and post-processed output of the LLM (e.g., probabilities, classification, unstructured text).	All	All	Supplemental
	6e	Provide details and rationale for any classification and, if applicable, how the probabilities were determined and thresholds identified.	All	C OF	NA
LLM Output	7a	Include metrics that capture the quality of generative outputs, such as consistency, relevance, and accuracy, compared to gold standards.	All	QA IR DG SS MT	7, 8
	7b	Report the outcome metrics' relevance to downstream task at deployment time and, where applicable, correlation of metric to human evaluation of the text for the intended use.	E H	All	7, 8
	7c	Clearly define the outcome, how the LLM predictions were calculated (e.g., formula, code, object, API), the date of inference for closed-source LLMs, and evaluation metrics.	E H	All	Supplemental
	7d	If outcome assessment requires subjective interpretation, describe the qualifications of the assessors, any instructions provided, relevant information on demographics of the assessors, and inter-assessor agreement.	All	All	Supplemental
	7e	Specify how performance was compared to other LLMs, humans, and other benchmarks or standards.	All	All	Supplemental
Annotation	8a	If annotation was done, report how text was labeled, including providing specific annotation guidelines with examples.	All	All	Supplemental
	8b	If annotation was done, report how many annotators labeled the dataset(s), including the proportion of data in each dataset that were annotated by more than 1 annotator, and the inter-annotator agreement.	All	All	Supplemental
	8c	If annotation was done, provide information on the background and experience of the annotators or characteristics of any models involved in labelling.	All	All	Supplemental
Prompting	9a	If research involved prompting LLMs, provide details on the processes used during prompt design, curation, and selection.	All	All	Supplemental
	9b	If research involved prompting LLMs, report what data were used to develop the prompts.	All	All	Supplemental
Summarization	10	Describe any preprocessing of the data before summarization.	All	SS	Supplemental
Instruction tuning/Alig	11	If instruction tuning/alignment strategies were used, what were the instructions, data, and interface used for evaluation,	M D	All	NA

nment		and what were the characteristics of the populations doing evaluation?			
Compute	12	Report compute, or proxies thereof (e.g., time on what and how many machines, cost on what and how many machines, inference time, floating-point operations per second (FLOPs)), required to carry out methods.	M D E	All	6
Ethical Approval	13	Name the institutional research board or ethics committee that approved the study and describe the participant-informed consent or the ethics committee waiver of informed consent.	All	All	6
Open Science	14a	Give the source of funding and the role of the funders for the present study.	All	All	2
	14b	Declare any conflicts of interest and financial disclosures for all authors.	All	All	2
	14c	Indicate where the study protocol can be accessed or state that a protocol was not prepared.	H	All	1
	14d	Provide registration information for the study, including register name and registration number, or state that the study was not registered.	H	All	1
	14e	Provide details of the availability of the study data.	All	All	1
	14f	Provide details of the availability of the code to reproduce the study results.	All	All	1
Public Involvemen t	15	Provide details of any patient and public involvement during the design, conduct, reporting, interpretation, or dissemination of the study or state no involvement.	H	All	1
Results					
Participants	16a	When using patient/EHR data, describe the flow of text/EHR/patient data through the study, including the number of documents/questions/participants with and without the outcome/label and follow-up time as applicable.	E H	All	Supplemental
	16b	When using patient/EHR data, report the characteristics overall and, for each data source or setting, and for development/evaluation splits, including the key dates, key characteristics, and sample size.	E H	All	Supplemental
	16c	For LLM evaluation that include clinical outcomes, show a comparison of the distribution of important clinical variables that may be associated with the outcome between development and evaluation data, if available.	E H	All	Table 1
	16d	When using patient/EHR data, specify the number of participants and outcome events in each analysis (e.g., for LLM development, hyperparameter tuning, LLM evaluation).	E H	All	Table 1
Performanc e	17	Report LLM performance according to pre-specified metrics (see item 7a) and/or human evaluation (see item 7d).	All	All	Table 3
LLM Updating	18	If applicable, report the results from any LLM updating, including the updated LLM and subsequent performance.	All	All	NA

Discussion					
Interpretation	19a	Give an overall interpretation of the main results, including issues of fairness in the context of the objectives and previous studies.	All	All	9-12
Limitations	19b	Discuss any limitations of the study and their effects on any biases, statistical uncertainty, and generalizability.	All	All	11
Usability of the LLM in context	19c	Describe any known challenges in using data for the specified task and domain context with reference to representation, missingness, harmonization, and bias.	E H	All	11
	19d	Define the intended use for the implementation under evaluation, including the intended input, end-user, level of autonomy/human oversight.	E H	All	9-12
	19e	If applicable, describe how poor quality or unavailable input data should be assessed and handled when implementing the LLM, i.e., what is the usability of the LLM in the context of current clinical care.	E H	All	11
	19f	If applicable, specify whether users will be required to interact in the handling of the input data or use of the LLM, and what level of expertise is required of users.	E H	All	11
	19g	Discuss any next steps for future research, with a specific view to applicability and generalizability of the LLM.	All	All	9-12

LLM = large language model; M = LLM methods; D = *de novo* LLM development; E = LLM evaluation; H = LLM evaluation in healthcare settings; C = classification; OF = outcome forecasting; QA = long-form question-answering; IR = information retrieval; DG = document generation; SS = summarization and simplification; MT = machine translation; EHR = electronic health record.

Note: For studies using existing LLMs, users should include reference(s) to reportable information if provided by the original developers or state that this information is not available.

Table 3
Table 3
