# Supplementary material for: Development and Validation of a Large Language Model Case Identification Strategy for Eosinophilic Esophagitis
Source: Gastro Hep Adv. 2026 Apr 16;5(7):100971. doi: 10.1016/j.gastha.2026.100971 (PMC13207557; doi:10.1016/j.gastha.2026.100971)
Supplement: Extended PDF [file mmc3.pdf]

Gastro Hep Advances 2026;5:100971

ORIGINAL RESEARCH—CLINICAL

Development and Validation of a Large Language Model Case Identification Strategy for Eosinophilic Esophagitis

Corey J. Ketchem,[1,2] Uğurcan Vurgun,[3] Agnes Wang,[4] Sunil Thomas,[5] Ashley Batugo,[5] John E. Pandolfino,[2] Gary W. Falk,[1] Kristle L. Lynch,[1] Evan S. Dellon,[6,7] Danielle L. Mowery,[3,5,§] and James D. Lewis[1,§]

[1]Division of Gastroenterology and Hepatology, Department of Medicine, Perelman School of Medicine, Hospital of the University of Pennsylvania, Philadelphia, Pennsylvania; [2]Division of Gastroenterology and Hepatology, Department of Medicine, Kenneth C. Griffin Esophageal Center of Northwestern Medicine, Feinberg School of Medicine, Northwestern University, Chicago, Illinois; [3]Department of Biostatistics, Epidemiology, & Informatics, University of Pennsylvania, Philadelphia, Pennsylvania; [4]Department of Computer and Information Science, School of Engineering & Applied Sciences, University of Pennsylvania, Philadelphia, Pennsylvania; [5]Institute for Biomedical Informatics, Perelman School of Medicine, University of Pennsylvania, Philadelphia, Pennsylvania; [6]Center for Esophageal Diseases and Swallowing, Division of Gastroenterology and Hepatology, Department of Medicine, University of North Carolina School of Medicine, Chapel Hill, North Carolina; and [7]Division of Gastroenterology and Hepatology, Department of Medicine, Center for Gastrointestinal Biology and Disease, University of North Carolina School of Medicine, Chapel Hill, North Carolina

BACKGROUND AND AIMS: Epidemiologic research in eosinophilic esophagitis (EoE) is limited by the accuracy and efficiency of case identification algorithms. We aimed to evaluate rule-based natural language processing (RB-NLP) and large language model–based natural language processing (LLM-NLP) pipelines for identifying EoE diagnoses and features from unstructured text. **METHODS:** We identified gastrointestinal pathology reports with any mention of "eosinophil" paired with gastroenterology clinic notes. Three hundred randomly selected patients were divided into training (n = 200, 56 with EoE) and testing (n = 100, 36 with EoE) sets. Manual chart review was the reference standard. RB-NLP used spaCy with medspaCy's clinical components; LLM-NLP prompts were developed through iterative human-in-the-loop refinement. In the validation set, we compared International Classification of Diseases (ICD) codes, RB-NLP, and LLM-NLP against the reference standard using sensitivity (recall), positive predictive value (precision), and F1 score. **RESULTS:** In the validation set, ICD codes alone had a sensitivity 0.86 (95% confidence interval [CI]: 0.75–0.97), a positive predictive value of 0.97 (95% CI: 0.91–1.0), and an F1 value of 0.91 (95% CI: 0.84–1.0). Combining ICD and LLM-assigned diagnosis yielded a 3-point improvement in F1 score (95% CI: −0.01 to 0.07; $P = .2$) compared to ICD alone. In a larger cohort (n = 580), the LLM + ICD approach identified the most EoE cases (n = 203) and captured 15% of cases missed by ICD codes. Clinical characteristics varied depending on the case identification strategy used. **CONCLUSION:** Combining LLM-NLP with a single ICD code reduced false negatives and modestly improved the F1 score compared to either method alone. This may represent a scalable approach to enhance EoE case identification in real-world data.

Keywords: Eosinophilic Esophagitis; Natural Language Processing; Artificial Intelligence; Electronic Health Records; Algorithms

Introduction

Eosinophilic esophagitis (EoE) is a chronic, allergen-mediated inflammatory disease that negatively impacts patients' quality of life and often follows a progressive course.[1,2] The incidence and prevalence of EoE have increased significantly over the past 15 years, leading to increased health-care cost and EoE-related emergency department visits.[3,4] These patterns highlight the need for high-quality research studies to address key knowledge gaps and inform strategies to mitigate the rising disease burden.[5] However, large-scale epidemiologic research for EoE is hampered by suboptimal accuracy of International Classification of Diseases (ICD) administrative codes, which suffer from both high false-positive and false-negative rates.[6–8] Although case identification algorithms requiring multiple ICD codes for EoE reduce false positives, such algorithms may exclude true cases, further impacting epidemiologic studies.

In clinical practice, a diagnosis of EoE requires integration of symptoms and histopathologic findings, much of which is documented in unstructured electronic health record (EHR) text. While manual chart review can be utilized for case

[§]Denotes co-senior authorship.

Abbreviations used in this paper: **EHR, electronic health record; EoE, eosinophilic esophagitis; ICD, International Classification of Diseases; LLM, large language model; LLM-NLP, large language model–based natural language processing; NLP, natural language processing; PPV, positive predictive value; RB-NLP, rules-based natural language processing.**

Most current article

© 2026 The Author(s). Published by Elsevier Inc. on behalf of American Gastroenterological Association Institute. This is an open access article under the CC BY license (http://creativecommons.org/licenses/by/4.0/).
2772-5723
https://doi.org/10.1016/j.gastha.2026.100971

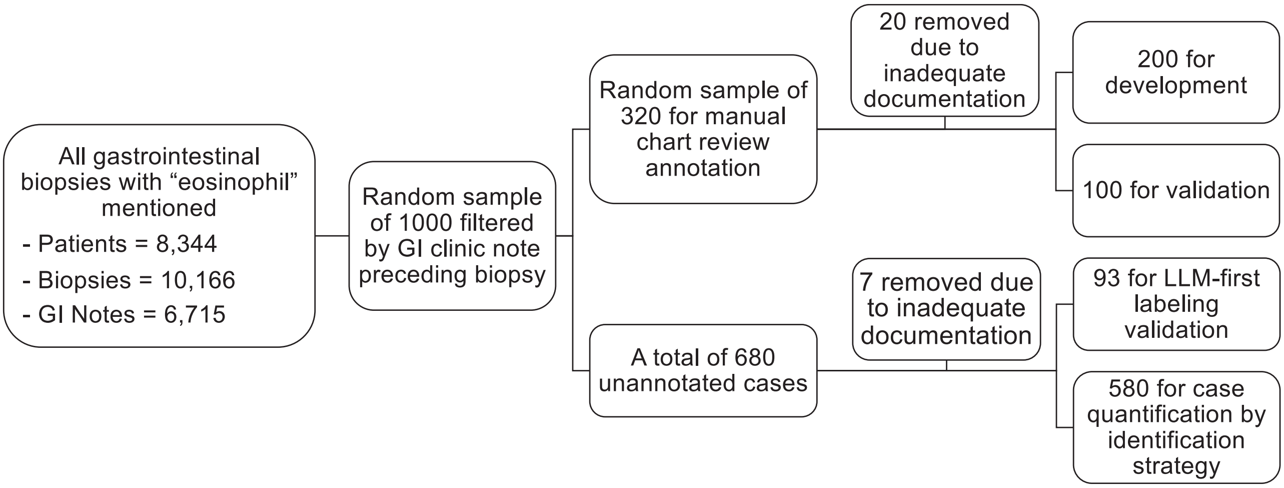

Figure 1. Cohort construction and annotation workflow for natural language processing pipeline.

identification, it is resource intensive, time consuming, and impractical at scale. Clinical natural language processing (NLP)—a field at the intersection of biomedicine, computer science, and artificial intelligence—offers a promising solution by enabling the extraction of key clinical concepts from unstructured EHR data. The traditional rule-based natural language processing (RB-NLP) approach has previously been evaluated in several gastrointestinal disease states.[9–11] However, recent advances in large language models (LLMs) have expanded NLP capabilities by enabling flexible, high-performance language understanding through natural language prompts, allowing information extraction without extensive programming or domain-specific linguistic expertise. This presents opportunities to standardize data extraction, minimize manual chart review, and process larger data volumes, with the goal of improving data quality and lowering cost.[12,13] As such, LLMs may prove valuable for diseases such as EoE, where diagnosis relies on integrating information from multiple sources.

We aimed to evaluate EoE case identification pipelines using RB-NLP and large language model–based natural language processing (LLM-NLP), benchmarked against a manually annotated reference standard. We hypothesized that LLM-NLP would improve recall (sensitivity) and precision (positive predictive value [PPV]) for identifying EoE cases and increase case yield when applied to a separate cohort.

Methods

Cohort Construction and Reference Standard

To develop the cohort, we queried Penn Medicine patients with clinical encounters from 2008 through April 2024, with patients initially identified by surgical pathology procedure orders (Figure 1). For each order, all associated "Final Diagnosis" unstructured text was aggregated, and records containing the term "eosinophil" were flagged regardless of the context; therefore, specimens with "no eosinophils" were also included. These were further filtered to include samples from relevant gastrointestinal anatomic sites (eg, esophagus, stomach, duodenum, colon, small intestine). Gastroenterology clinical encounters 1 year before or after the pathology were identified, and the full unstructured clinical text from progress notes was aggregated. "Inadequate documentation" was defined as clinic note text that lacked sufficient symptom information to assess for EoE diagnostic criteria and were removed during manual review for development and validation. All data were deidentified using Philter, an open source deidentification tool.[14] Demographics and ICD codes for EoE (all converted to ICD-10, K20.0) were collected from all available time points, while age was at first biopsy.

Diagnostic classification for both the reference standard (manual review by C.J.K.) and NLP-based approaches followed a predefined decision algorithm. Patients were classified as having EoE if either of the following criteria were met: (1) a prior diagnosis of EoE was identified in the clinic note and not negated; or (2) pathology features extracted from the pathology report met histologic criteria for EoE ($\geq$15 eosinophils per high-power field) in conjunction with esophageal dysfunction symptoms extracted from the clinic note.

For both NLP methods used, we included patients with at least 1 associated clinical note between 1 and 60 days prior to the corresponding biopsy date (Supplementary Methods). This window was chosen to balance capturing clinically relevant symptoms that reflected the prebiopsy context while minimizing inclusion of outdated or postdiagnostic documentation that could introduce bias. Subjects were randomly allocated into development ($n = 200$; 56 with EoE) and validation ($n = 100$; 36 with EoE) sets. Assuming a PPV of 80%, the sample sizes yielded 95% confidence intervals (CIs) with margins of error ranging from $\pm$9% to $\pm$11%.

Algorithms for EoE Case Identification

We examined 3 different approaches to EoE case identification. The ICD-only identification strategy was defined as having ever received at least 1 EoE-related ICD code during the available observation period.

The RB-NLP pipeline analyzed the unstructured pathology and clinic notes using spaCy, with medspaCy's clinical components enabled (Supplementary Methods).[15] Sentence segmentation was performed using a custom boundary component followed by spaCy's sentencizer to improve parsing of structured

clinical text. We implemented curated TargetRule definitions with medSpaCy's TargetMatcher to identify key EoE-related concepts (Supplementary Table 1). Classification as a positive diagnosis of EoE was the same as previously mentioned, termed "RB-NLP diagnosis." The "RB + ICD diagnosis" classified patients as positive if they ever had an EoE-related ICD code and/or met RB-NLP diagnosis. All features were evaluated at the document level, and no machine learning classifier was used. All features and diagnoses were compared to the reference standard.

The LLM-NLP pipeline utilized OpenAI GPT-4-turbo-128k, deployed using the secure and Health Insurance Portability and Accountability Act-compliant Microsoft Azure Databricks infrastructure within Penn Medicine's tenant (Supplementary Methods). We implemented a structured prompt engineering approach that instructed the model to extract diagnostic variables that were used to assign an EoE diagnosis, which were then mapped to diagnosis using a prespecified rules-based decision layer. Supplementary Table 1 summarizes the primary clinical concepts extracted from the notes, including dysphagia, food impaction, reflux symptoms, eosinophil presence, eosinophil count per high-power field, and prior history of EoE, among others. This framework defined EoE based on either a documented history of EoE or concordant symptom and pathology findings ("LLM-features diagnosis"). In parallel, the model was prompted to independently assign an EoE diagnosis based on the clinic and pathology text alone, without reliance on extracted features ("LLM-assigned diagnosis"). Finally, the "LLM + ICD diagnosis" approach classified patients as positive if they ever had an EoE-related ICD code and/or met the LLM-assigned diagnosis criteria. During development, a human-in-the-loop approach was used for iterative prompt refinement (Supplementary Figure 1; Supplementary Methods).[16] Once finalized, the prompt was locked, and performance was evaluated in an independent validation cohort.

Statistical Analysis

We evaluated the performance of ICD alone, RB-NLP (±ICD), and LLM-NLP (±ICD) for identifying EoE cases against the manually annotated reference standard. Model performance was assessed using the following binary classification metrics: accuracy, precision (PPV), recall (sensitivity), specificity, NPV, F1 score (harmonic mean between recall and precision), and Cohen kappa. Numeric value extraction (eg, eosinophil counts) was assessed using linear correlation (r^2). To estimate 95% CIs, we applied nonparametric bootstrap resampling (1000 replicates). Statistical significance was determined based on whether the bootstrap-derived 95% CI for the difference compared to the referent excluded zero. McNemar chi-square test was used to compare paired proportions, while chi-square tests evaluated the agreement across case identification methods. For the LLM-NLP approach, time and cost of implementation were estimated. All statistical analyses were performed using Stata (version 18.0; StataCorp) and Python (version 3.12.3; Python Software Foundation), with the latter used for NLP programming. This study was approved by the University of Pennsylvania Institutional Review Board.

Results

Development and Validation Cohort Demographics

A random sample of 300 patients was divided into 200 for the development and 100 for the validation cohorts. The median age at biopsy was 52.0 years (interquartile range [IQR]: 41.0–64.0), with younger patients in the validation cohort (49.5 years) compared to the development cohort (53.5 years) (Table 1). Overall, 54% of the cohort was female, with a higher proportion in the development cohort (58%) than in the validation cohort (46%). The majority of patients identified as White (72%), followed by Black (22%), with racial distribution differing between cohorts (White: 66% vs 85%; Black: 28.5% vs 8% in development vs validation, respectively). A total of 92 patients (30.7%) met the manually annotated reference standard for EoE diagnosis, with a higher proportion in the validation cohort (36%) than in the development cohort (28%).

ICD-Only Performance

Among the validation cohort, the ICD-only method yielded precision (PPV) of 0.97 (0.91–1.00) and accuracy of 0.94 (0.89–1.00), with moderate recall (0.86 [0.75–0.97]) and kappa of 0.87 (0.76–0.97), consistent with an F1 score of 0.91 (0.84–1.00) (Table 2).

RB-NLP Performance

The RB-NLP approach achieved balanced precision (PPV) and recall (sensitivity) (both 0.75 [0.61–0.89]), with an accuracy of 0.82 (0.75–0.90) and kappa of 0.61 (0.45–0.77) (Supplementary Table 2). The combined RB + ICD method demonstrated the highest recall (sensitivity) (0.89 [0.79–0.99]) and strong overall performance, with an F1 score of 0.83 (0.73–0.93), accuracy of 0.87 (0.80–0.94), and Cohen kappa of 0.73 (0.59–0.86).

Regarding the performance metrics for individual clinical and histologic variables, the validation dataset revealed that positive eosinophils, eosinophil enumeration, dysphagia, and reflux symptoms were extracted with high precision (PPV >0.85) and F1 scores (all >0.75) (Supplementary Figure 2). Variables such as food impaction and descriptive increases in eosinophils showed lower precision, highlighting variability in extraction performance by concept type. Regarding numeric extraction of peak eosinophil count, the RB-NLP system showed moderate correlation in the validation cohort ($R^2 = 0.48$) (Supplementary Figure 3).

LLM-NLP Performance

In the validation cohort, the combined LLM + ICD method showed the highest overall performance, consistent with the development cohort (Table 2). It achieved a recall of 0.92 (0.83–1.00), precision of 0.97 (0.91–1.00), and accuracy of 0.96 (0.92–1.00), with a Cohen kappa of 0.91 (0.83–1.00) and F1 score of 0.94 (0.89–1.00). Notably, the LLM-assigned diagnosis achieved perfect precision and specificity (1.00), though at the cost of lower recall (0.75 [0.61–0.89]). When compared to the ICD-only referent, both LLM-derived features and LLM-assigned diagnosis had slightly lower F1 scores (ΔF1: −0.05 [95% CI: −0.13 to 0.04] and −0.05 [95% CI: −0.16 to 0.05], respectively),

Table 1. Demographics and Clinical Characteristics of the Development and Validation Cohorts

Variable	Total cohort (N = 300)	Development cohort (N = 200)	Validation cohort (N = 100)
Demographics, n (%)			
Age at biopsy	52.0 (41.0–64.0)	53.5 (42.0–64.0)	49.5 (39.0–63.5)
Female	161 (54)	115 (58)	46 (46)
Male	139 (46)	85 (43)	54 (54)
Race, n (%)			
Asian	4 (1)	2 (1.0)	2 (2)
Black or African American	65 (22)	57 (29)	8 (8)
Multiracial	1 (0.3)	1 (0.5)	0 (0)
Patient declined	1 (0.3)	1 (0.5)	0 (0)
Some other race	7 (2)	3 (1.5)	4 (4)
Unknown	6 (2)	5 (2.5)	1 (1)
White	216 (72)	131 (66)	85 (85)
Ethnicity, n (%)			
Hispanic Latino	8 (3)	5 (3)	3 (3)
Not Hispanic or Latino	292 (97)	195 (98)	97 (97)
Histology, n (%)			
Positive eosinophils	220 (73)	146 (73)	74 (74)
Esophageal localization of eosinophils	162 (54)	108 (54)	54 (54)
Eosinophil counts increased (≥15 eos/hpf)	103 (34)	59 (30)	44 (44)
Eosinophil count	20.0 (6.0–50.0)	14.0 (5.5–48.0)	24.0 (8.0–59.0)
Symptoms and history, n (%)			
Dysphagia	154 (51)	108 (54)	46 (46)
Food impaction	57 (19)	45 (23)	12 (12)
Reflux symptoms	182 (61)	131 (66)	51 (51)
Documented past EoE history	56 (19)	32 (16)	24 (24)
Reference standard EoE diagnosis	92 (31)	56 (28)	36 (36)
EoE diagnostic code	81 (27)	49 (25)	32 (32)

Histology and symptom features extracted via manual annotation.

whereas the combined LLM + ICD method demonstrated a modest but favorable improvement (ΔF1: +0.03 [95% CI: −0.01 to 0.07]). Comparing NLP approaches, the LLM + ICD method showed an 11% higher F1 score than RB + ICD (difference: 0.11 [95% CI: 0.04–0.19]; *P* = .004).

To validate the real-world performance of the LLM-NLP in the absence of prelabeled data, we first allowed the model to assign EoE diagnoses on a cohort of 93 patients (7 removed due to inadequate clinical notes) and subsequently verified its predictions against a manual review (Supplementary Methods). The combination of ICD codes and LLM diagnosis achieved the highest recall at 0.93 (0.84–1.00) and F1 score of 0.92 (0.84–0.99), suggesting feasibility of an LLM-first labeling strategy (Supplementary Tables 3 and 4).

LLM-NLP extraction of individual variables displayed strong performance within the development and validation datasets (Figure 2). Only food impaction had a PPV <0.80, while most F1 scores were >0.70, suggesting a balanced trade-off between precision and recall. Regarding numeric extraction of peak eosinophil count, the LLM-NLP system showed moderate correlation (R^2 = 0.47) (Supplementary Figure 3).

Comparison of Case Identification Strategies

Because the LLM-NLP strategies demonstrated high performance metrics and validity for an LLM-first labeling strategy, we applied these methods to the remaining unannotated cohort (n = 580) to simulate a larger real-world data set (Supplementary Methods). The LLM + ICD approach identified the highest number (n = 203) of EoE cases, significantly more than either ICD-only (n = 173; *P* < .001) or LLM-NLP-only (n = 158; *P* < .001) (Figure 3). Of the 203 patients identified by the LLM + ICD method, 85% (n = 173) had an ICD code, while 15% (n = 30) were missed by ICD alone. Difference between identification strategies included LLM + ICD cases being younger (median age, 31.0 years [IQR: 26.0–38.0]; *P* = .01), with LLM-NLP only having more dysphagia (87%; *P* < .001) and food impactions (40%; *P* < .001) (Table 3).

LLM-NLP Time and Cost Estimations

The initial pipeline development required approximately 40 hours for EHR data query and 40–60 hours of programmer time, including prompt engineering (15–20 hours), infrastructure setup (10–15 hours), batch processing implementation (10–15 hours), and evaluation framework development (10–15 hours). Iterative prompt refinement added 20 hours, for a total of 60–80 hours for pipeline development. Processing 50 clinical records with detailed clinical and biopsy reports (1866.0 tokens [IQR: 1305.5–2549.3] per note) consumed approximately $25–$40 in application programming interface costs using

Table 2. LLM-Based NLP Development and Validation Performance Metrics, Reported as Point Estimates With 95% Confidence Intervals (CI)

Method	Precision (PPV)	Recall (sensitivity)	Specificity	NPV	Accuracy	Kappa	F1	ΔF1 Bootstrap
Development set								
ICD alone	0.86 [0.76–0.96]	0.75 [0.64–0.86]	0.95 [0.92–0.99]	0.91 [0.86–0.95]	0.90 [0.85–0.94]	0.73 [0.62–0.84]	0.80 [0.71–0.89]	–
LLM-features diagnosis	0.85 [0.75–0.94]	0.88 [0.79–0.96]	0.94 [0.90–0.98]	0.95 [0.92–0.99]	0.92 [0.88–0.96]	0.80 [0.71–0.90]	0.86 [0.79–0.93]	–
LLM-assigned diagnosis	0.92 [0.84–1.0]	0.80 [0.70–0.91]	0.97 [0.95–1.0]	0.93 [0.89–0.97]	0.93 [0.89–0.96]	0.81 [0.71–0.90]	0.86 [0.78–0.93]	–
ICD + LLM diagnosis	0.85 [0.76–0.94]	0.91 [0.84–0.99]	0.94 [0.90–0.98]	0.96 [0.93–1.0]	0.93 [0.90–0.97]	0.83 [0.74–0.92]	0.88 [0.81–0.95]	–
Validation set								
ICD alone	0.97 [0.91–1.0]	0.86 [0.75–0.97]	0.98 [0.95–1.0]	0.93 [0.86–1.0]	0.94 [0.89–1.0]	0.87 [0.76–0.97]	0.91 [0.84–1.0]	Referent
LLM-derived features	0.93 [0.85–1.0]	0.81 [0.68–0.94]	0.97 [0.91–1.0]	0.90 [0.83–0.97]	0.91 [0.85–0.97]	0.79 [0.67–0.92]	0.87 [0.77–0.96]	−0.05 [−0.13 to 0.04]
LLM-assigned diagnosis	1.0 [1.0–1.0]	0.75 [0.61–0.89]	1.0 [1.0–1.0]	0.87 [0.80–0.95]	0.91 [0.85–1.0]	0.79 [0.66–0.92]	0.86 [0.77–0.95]	−0.05 [−0.16 to 0.05]
ICD + LLM diagnosis	0.97 [0.91–1.0]	0.92 [0.83–1.0]	0.98 [0.95–1.0]	0.95 [0.90–1.0]	0.96 [0.92–1.0]	0.91 [0.83–1.0]	0.94 [0.89–1.0]	0.03 [−0.01 to 0.07]

In the validation set, the combined ICD + LLM approach demonstrated a numeric improvement in F1 score compared with ICD alone ($P = .20$). Sensitivity was also numerically higher for ICD + LLM vs ICD alone ($P = .15$).

GPT-4, translating to roughly $0.50–$0.80 per patient record. For a cohort of 1000 patients, total computational costs would approximate $500–$800. The automated pipeline achieved a processing rate of 10–12 seconds per record (estimated 2–4 hours for 1000 patients), compared to manual chart review by a trained clinician requiring 15–20 minutes per record (250–333 hours per 1000 patients), representing a more rapid approach.

Discussion

Accurate case identification is critical for limiting bias and enabling high-quality research studies with real-world data.[17] Many studies on EoE have relied on diagnostic code algorithms, yet this approach can misclassify or fail to capture true cases. Herein, we demonstrate an internally validated and accurate approach using LLM-based NLP techniques that integrate clinical and histologic information, achieving high precision (PPV) that limits false positives. When combined with a single ICD code, the LLM-NLP approach has additional gains in recall (sensitivity), reducing the false negatives with minimal sacrifice to precision. Additionally, the LLM-NLP allows simultaneous extraction of individual variables with a low false-positive rate. Furthermore, when applied to a separate, larger sample, the LLM + ICD pipeline identified additional EoE cases with differences in baseline symptoms and characteristics by identification strategy, suggesting systematic variation between approaches.

Despite the importance of accurate case identification, validated algorithms for EoE remain limited and with notable shortcomings. Both adult and pediatric studies have reported high specificity and low sensitivity, indicating that more than half of cases were missed when relying only on the ICD code.[6,8] The aforementioned adult study found differences in clinical characteristics between true positive and false negatives, suggesting potential for selection bias with ICD code reliance. To address challenges with precision, some have proposed more restrictive algorithms, requiring multiple ICD or procedure codes.[7] While this approach increases precision (PPV), there is a risk of excluding true cases labeled with only a single code, thereby lowering sample size and potentially selecting for patients with more persistent symptoms. The current study revealed that a combination LLM + ICD method reduced false negatives and relied only on a single ICD code without the trade-off of overly restrictive criteria. Moreover, the demographic and clinical differences between identification methods in our study represent a central finding that could have implications for observational research. The LLM-NLP approach identified individuals near the upper limit of the peak incidence age range, which may be related to clinicians more frequently considering alternative diagnoses instead of EoE in older patients. Furthermore, the higher frequency of dysphagia and food impactions in this group may suggest phenotypic variation, whereas individuals identified only by ICD code appeared more often asymptomatic and in histologic

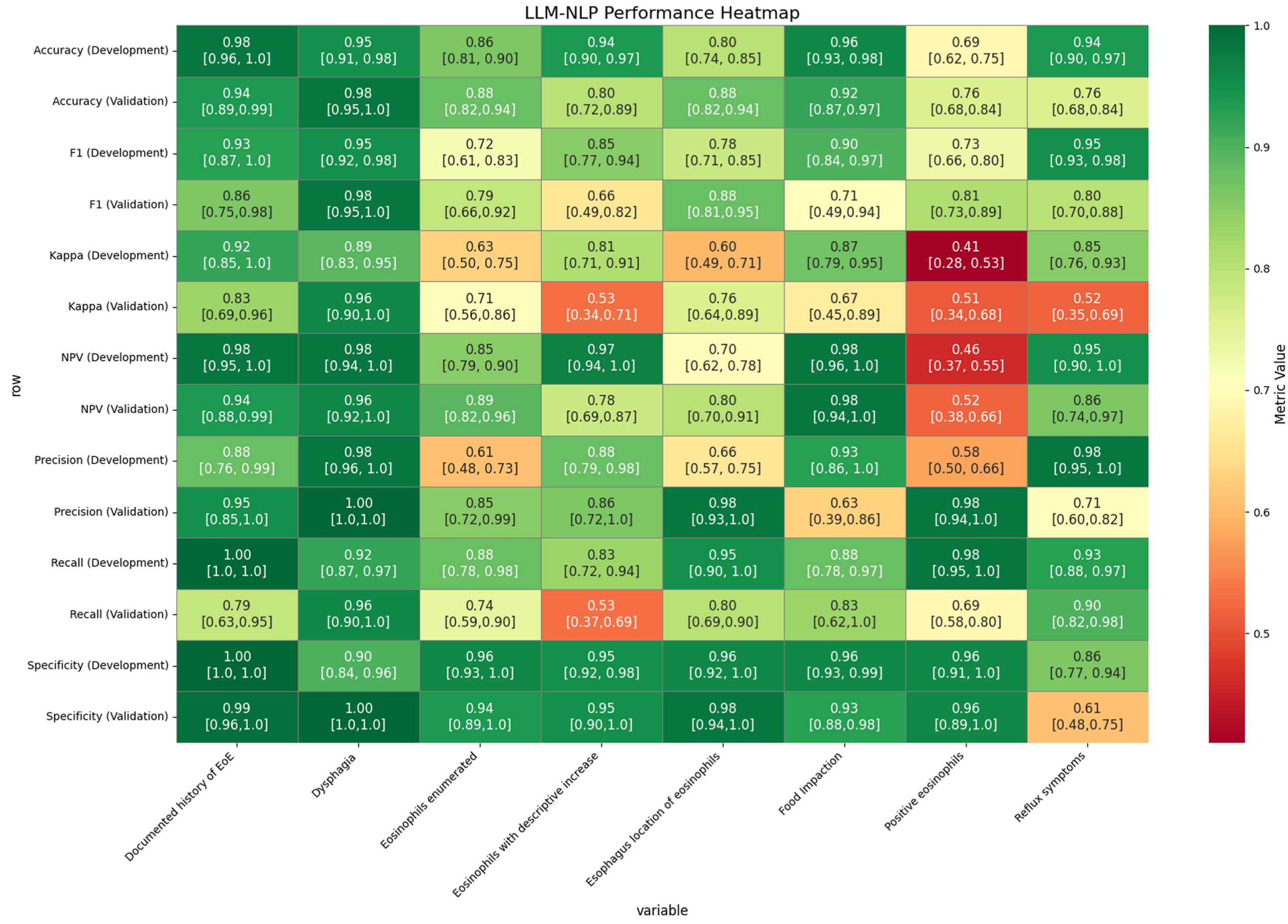

Figure 2. Heatmap of LLM-based NLP development and validation performance metrics for individual variables, point estimates with 95% confidence intervals. Metric values are color-coded from green (high performance) to red (low performance).

remission. These findings warrant further investigation but highlight the potential for selection bias depending on the case ascertainment method, as well as the potential added value of LLM-NLP in improving case identification.

Interest in applying LLMs in gastroenterology is growing.[18,19] While RB-NLP systems have been widely used for various task,[20] their broader adoption has been limited by the programming and linguistic expertise required for implementation. To date, relatively few studies have leveraged LLMs specifically for disease case identification, and even fewer have focused on EoE. One study showed mixed accuracy of LLM responses to patient-oriented questions about EoE.[21] Studies in other gastrointestinal conditions have demonstrated that LLM-based NLP pipelines can accurately identify conditions such as gastrointestinal bleeding using nursing notes and laboratory data, demonstrating low false-positive and false-negative rates.[22] Additional work has shown LLMs capable of extracting histopathologic features from colorectal specimens with high precision and recall.[23] Others have evaluated LLM-based approaches for identifying features of cirrhosis and hepatocellular carcinoma, with authors proposing that LLM-NLP could serve as a sufficient reference standard in lieu of manual annotation.[24,25] Building on this prior work, our study demonstrates accurate case identification of EoE using LLM-NLP. We observed comparable performance of an LLM-first approach, supporting its potential as a scalable alternative to traditional manual review. Beyond case identification, our LLM-NLP pipeline also extracted individual diagnostic features with high precision, further demonstrating its potential to enhance or replace manual extraction. Another key innovation of our study is the integration of both clinical and pathology text to assign diagnosis for a clinicopathologic disease. To our knowledge, this represents one of the first applications of LLMs to integrate these text data types and supports the use of LLMs for accurate case ascertainment from real-world data, with or without traditional code-based methods.

Beyond the test characteristics of the NLP approaches, several observations and potential limitations warrant discussion. First, ICD coding for EoE demonstrated higher performance in our cohort than previously reported. When baseline precision is high, as observed here, improvements in recall may yield only modest changes in performance despite identifying additional true cases. This likely reflects institutional expertise and cohort construction, as individuals with ICD codes lacking confirmatory pathology may have been excluded, reducing false positives and potentially limiting

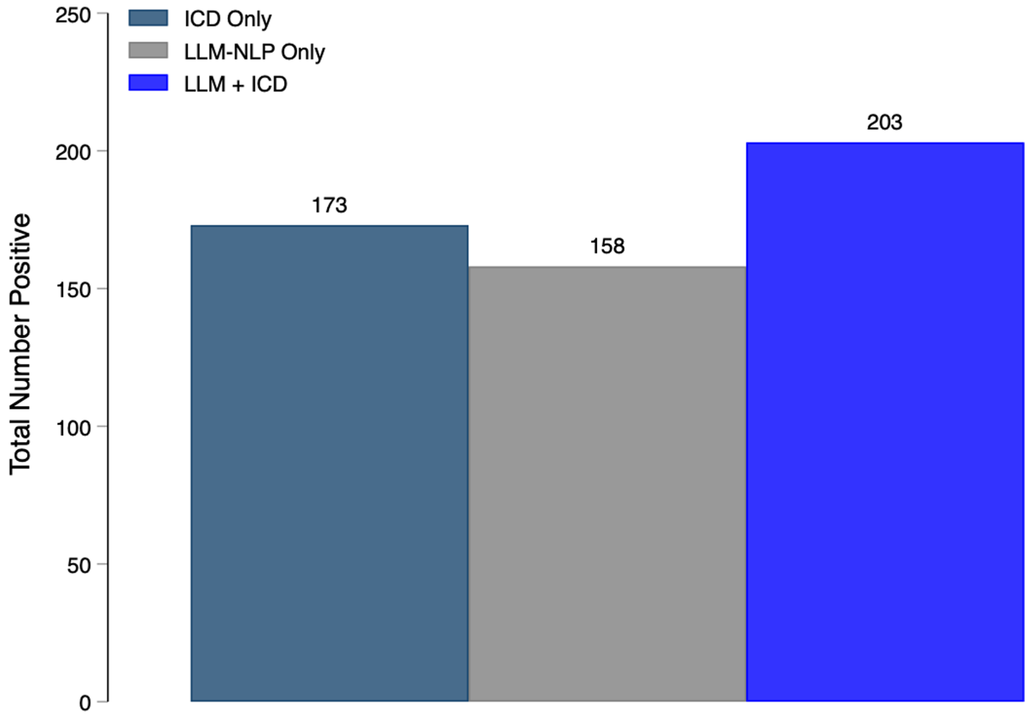

Figure 3. Total number of EoE patients identified by ICD alone, LLM-NLP, and LLM + ICD. LLM + ICD identified more cases than ICD alone ($P < .001$) and LLM-NLP alone ($P < .001$), while there was no significant difference between ICD alone and LLM-NLP alone ($P = .08$).

Table 3. Clinical Characteristics Based on Identification Method

	ICD only (N = 45)	LLM-NLP only (N = 30)	LLM + ICD (N = 128)	No diagnosis (N = 376)	*P*
Demographics, n (%)					
Age at biopsy, median (IQR)	34.0 (27.0–46.0)	36.5 (30.0–49.0)	31.0 (26.0–38.0)	43.5 (29.0–60.0)	.01
Female	17 (38%)	11 (37%)	44 (34%)	201 (54%)	.90
Male	28 (62%)	19 (63%)	84 (66%)	175 (47%)	
Race, n (%)					
Asian	1 (2%)	0 (0%)	8 (6%)	18 (5%)	.50
Black or African American	3 (7%)	5 (17%)	10 (8%)	41 (11%)	
Multi	0 (0%)	0 (0%)	1 (1%)	4 (1%)	
Patient declined	0 (0%)	0 (0%)	0 (0%)	1 (0.3%)	
Other race	0 (0%)	0 (0%)	2 (2%)	14 (4%)	
Unknown	0 (0%)	0 (0%)	0 (0%)	19 (5%)	
White	41 (91%)	25 (83%)	107 (84%)	277 (74%)	
Ethnicity, n (%)					
Hispanic Latino	0 (0%)	0 (0%)	1 (1%)	16 (4%)	.10
Not Hispanic or Latino	43 (96%)	30 (100%)	127 (99%)	357 (95%)	
Histology, n (%)					
Positive eosinophils	34 (76%)	30 (100%)	119 (93%)	138 (37%)	<.001
Eosinophils located in esophagus	34 (76%)	30 (100%)	119 (93%)	91 (24%)	<.001
Eosinophil counts increased (≥15 eos/hpf)	26 (58%)	29 (97%)	103 (81%)	19 (5%)	<.001
Eosinophil count	20.0 (10.0–48.5)	25.0 (20.0–32.0)	30.0 (17.0–70.0)	5.0 (2.0–10.0)	.30
Symptoms and history, n (%)					
Dysphagia	15 (33%)	26 (87%)	102 (80%)	132 (35%)	<.001
Food impaction	8 (18%)	12 (40%)	49 (38%)	29 (8%)	.03
Reflux symptoms	19 (42%)	20 (67%)	78 (61%)	236 (63%)	.05
Documented past EoE history	1 (2%)	5 (17%)	87 (68%)	0 (0%)	<.001
EoE diagnostic code	45 (100%)	0 (0%)	128 (100%)	0 (0%)	<.001

Histology and symptom features are LLM-NLP assigned. Statistical testing excludes the “no diagnosis” column.

generalizability to cohorts constructed differently. However, this does not diminish the utility of the LLM-NLP approach, which reduced false positives while identifying additional cases missed by ICD alone. Prior studies support the robustness of LLM-based approaches across settings where ICD performance varies, but more testing is required to address this with our approch.[26]

Second, extraction of individual variables showed differences between methods, where LLM-NLP generally outperformed RB-NLP, though RB-NLP performed well for highly structured tasks. This aligns with prior work showing superior performance of LLM-NLP for symptom extraction,[27] though others have proposed advantages to a hybrid approach.[22] More testing is necessary to clarify this matter. Third, our analysis was restricted to the prebiopsy clinic notes to preserve temporality and due to computational and token constraints (ie, limits on the amount of text that can be processed), limiting review of the full patient record (eg, general medicine or allergy/immunology clinics). Inclusion of all documents could have enhanced performance, and external records might have strengthened generalizability. At present, broader application of LLM-NLP approaches face practical limitations, but studies are underway to understand multisite applicability. Fourth, we used an off-the-shelf LLM (OpenAI models), which simplified implementation, but alternative models may offer better performance, cost, or explainability. Lastly, the reference standard was annotated by a single reviewer, which may introduce misclassification bias; however, LLM-based case ascertainment is an evolving area, and future studies will examine multireviewer annotation and formal interrater agreement to strengthen validation. Ongoing efforts to address these limitations will advance the use of LLMs for real-world data extraction.

Several strengths of this study are worth emphasizing. We introduce an accurate approach using LLM-based NLP that has potential to enhance or replace code-based methods for cohort identification while enabling the simultaneous extraction of the individual diagnostic variables. Application of NLP methods within secure computational environments, coupled with structured prompt development, ensured robustness and consistency of data extraction. Additionally, the human-in-the-loop approach allows for human oversight with ongoing refinement, supporting adaptable, goal-specific applications.[16,28] We acknowledge demographic imbalance between the development and validation cohorts; however, this may be considered a strength since clinical features often vary across populations and sites. Additionally, while the F1 increase for LLM + ICD over ICD-only was modest, the LLM-NLP method captured 30 additional EoE cases (15% increase vs ICD alone) in the larger sample, demonstrating that small metric gains in validation can yield meaningful improvements in real-world case identification. The impact of such gains is likely to increase as these methods scale to larger datasets. Finally, we highlight the cost and time required to implement LLM-NLP pipelines, an important yet underexplored consideration in the current literature. Some studies have demonstrated cost benefits, particularly with multiple queries per document, similar to our approach.[13,29] While further research is needed to define cost-to-benefit tradeoffs, our approach suggests that, for large-scale studies, months of manual annotation work would be reduced to days of automated processing, with the primary time investment shifted to quality assurance and validation rather than primary data extraction.

Conclusion

We developed and validated an LLM-based NLP approach, revealing that the LLM-NLP pipeline has high performance metrics for identifying EoE patients and extracting diagnostic variables. This approach offers the capability for consistent, large-scale data extraction, potentially reducing both manual effort and associated costs. When applied to a larger cohort, the LLM-NLP method identified additional cases, revealing clinical differences based on identification strategy. Combining LLM-NLP with a single diagnostic code offers a scalable and accurate alternative to traditional code-based algorithms while enabling simultaneous extraction of individual diagnostic variables. These tools have the potential to enhance real-world data pipelines, improve data quality, and advance research in EoE and other clinicopathologic conditions. Further work is needed to evaluate scalability and generalizability across diverse data sources, diseases, and health-care settings.

Supplementary Materials

Material associated with this article can be found, in the online version, at https://doi:10.1016/j.gastha.2026.100971.

References

1. Ketchem CJ, Starling AS. Insights into the natural history and disease course of eosinophilic esophagitis. Ann Allergy Asthma Immunol 2025;135:155–161.
2. van Klink ML, Bredenoord AJ. Health-related quality of life in patients with eosinophilic esophagitis. Immunol Allergy Clin North Am 2024;44(2):265–280.
3. Thel HL, Anderson C, Xue AZ, et al. Prevalence and costs of eosinophilic esophagitis in the United States. Clin Gastroenterol Hepatol 2025;23(2):272–280.e8.
4. Lam AY, Lee JK, Coward S, et al. Epidemiologic burden and projections for eosinophilic esophagitis-associated emergency department visits in the United States: 2009-2030. Clin Gastroenterol Hepatol 2023; 21(12):3041–3050.e3.
5. Bredenoord AJ, Patel K, Schoepfer AM, et al. Disease burden and unmet need in eosinophilic esophagitis. Am J Gastroenterol 2022;117(8):1231–1241.
6. Rybnicek DA, Hathorn KE, Pfaff ER, et al. Administrative coding is specific, but not sensitive, for identifying eosinophilic esophagitis. Dis Esophagus 2014;27(8):703–708.
7. Low EE, Song Q, Yadlapati R, et al. Development and validation of the Veterans Affairs Eosinophilic

Esophagitis Cohort. Clin Gastroenterol Hepatol 2023;21(12):3030–3040.e4.
8. Robson J, Korgenski K, Parsons K, et al. Sensitivity and specificity of administrative medical coding for pediatric eosinophilic esophagitis. J Pediatr Gastroenterol Nutr 2019;69(2):e49–e53.
9. Nguyen Wenker T, Natarajan Y, Caskey K, et al. Using natural language processing to automatically identify dysplasia in pathology reports for patients with Barrett's esophagus. Clin Gastroenterol Hepatol 2023;21(5):1198–1204.
10. Stidham RW, Yu D, Zhao X, et al. Identifying the presence, activity, and status of extraintestinal manifestations of inflammatory bowel disease using natural language processing of clinical notes. Inflamm Bowel Dis 2023;29(4):503–510.
11. Chang EK, Yu CY, Clarke R, et al. Defining a patient population with cirrhosis: an automated algorithm with natural language processing. J Clin Gastroenterol 2016;50(10):889–894.
12. Goldberg SI, Niemierko A, Turchin A. Analysis of data errors in clinical research databases. AMIA Annu Symp Proc 2008;2008:242–246.
13. Huang J, Yang DM, Rong R, et al. A critical assessment of using ChatGPT for extracting structured data from clinical notes. NPJ Digit Med 2024;7(1):106.
14. Norgeot B, Muenzen K, Peterson TA, et al. Protected Health Information filter (Philter): accurately and securely de-identifying free-text clinical notes. NPJ Digit Med 2020;3:57.
15. Eyre H, Chapman AB, Peterson KS, et al. Launching into clinical space with medspaCy: a new clinical text processing toolkit in Python. AMIA Annu Symp Proc 2021;2021:438–447.
16. Wang ZJ, Choi D, Xu S, et al. Putting humans in the natural language processing loop: a survey. arXiv 2021;arXiv:210304044.
17. Weinstein EJ, Ritchey ME, Lo Re V 3rd. Core concepts in pharmacoepidemiology: validation of health outcomes of interest within real-world healthcare databases. Pharmacoepidemiol Drug Saf 2023;32(1):1–8.
18. Shahab O, El Kurdi B, Shaukat A, et al. Large language models: a primer and gastroenterology applications. Ther Adv Gastroenterol 2024;17:17562848241227031.
19. Giuffre M, Kresevic S, Pugliese N, et al. Optimizing large language models in digestive disease: strategies and challenges to improve clinical outcomes. Liver Int 2024;44(9):2114–2124.
20. Stammers M, Ramgopal B, Owusu Nimako A, et al. A foundation systematic review of natural language processing applied to gastroenterology & hepatology. BMC Gastroenterol 2025;25(1):58.
21. Ketchem CJ, Lynch KL, Chang JW, et al. Artificial intelligence chatbot shows multiple inaccuracies when responding to questions about eosinophilic esophagitis. Clin Gastroenterol Hepatol 2024;22(5):1133–1135.
22. Zheng NS, Keloth VK, You K, et al. Detection of gastrointestinal bleeding with large language models to aid quality improvement and appropriate reimbursement. Gastroenterology 2025;168(1):111–120.e4.
23. Johnson B, Bath T, Huang X, et al. Large language models for extracting histopathologic diagnoses of colorectal cancer and dysplasia from electronic health records. medRxiv 2025;2024.11.27.24318083.
24. Far AT, Bastani A, Lee A, et al. Evaluating the positive predictive value of code-based identification of cirrhosis and its complications utilizing GPT-4. Hepatology 2025;81(6):1753–1763.
25. Ge J, Li M, Delk MB, et al. A comparison of a large language model vs manual chart review for the extraction of data elements from the electronic health record. Gastroenterology 2024;166(4):707–709.e3.
26. Sun VH, Heemelaar JC, Hadzic I, et al. Enhancing precision in detecting severe immune-related adverse events: comparative analysis of large language models and international classification of disease codes in patient records. J Clin Oncol 2024;42(35):4134–4144.
27. Patel PV, Davis C, Ralbovsky A, et al. Large language models outperform traditional natural language processing methods in extracting patient-reported outcomes in inflammatory bowel disease. Gastro Hep Adv 2025;4(2):100563.
28. De Angeli K, Gao S, Alawad M, et al. Deep active learning for classifying cancer pathology reports. BMC Bioinformatics 2021;22(1):113.
29. Klang E, Apakama D, Abbott EE, et al. A strategy for cost-effective large language model use at health system-scale. NPJ Digit Med 2024;7(1):320.

Received December 26, 2025. Accepted April 10, 2026.

Correspondence:
Address correspondence to: Corey J. Ketchem, MD, Division of Gastroenterology, Department of Medicine, Northwestern University Feinberg School of Medicine, 211 East Ontario #1700, Office WS-G, Chicago, Illinois 60611.

Authors' Contributions:
Corey J. Ketchem: Planning and conducting the study, collecting and interpreting data, and drafting the article. Uğurcan Vurgun: Collecting and interpreting data and critical revision of the article. Agnes Wang: Collecting and interpreting data and critical revision of the article. Sunil Thomas: Collecting data and critical revision of the article. Ashley Batugo: Collecting data and critical revision of the article. John E. Pandolfino: Interpreting data and critical revision of the article. Gary W. Falk: Planning the study, interpreting data, and critical revision of the article. Evan S. Dellon: Planning the study, interpreting data, and critical revision of the article. Kristle L. Lynch: Planning the study, interpreting data, and critical revision of the article. Danielle L. Mowery: Planning and conducting the study, interpreting data, and critical revision of the article. James D. Lewis: Planning and conducting the study, interpreting data, and critical revision of the article. All authors approved the final article.

Conflicts of Interest:
This author discloses the following: Kristle L. Lynch: Consultant for Sanofi/Regeneron, Takeda, and Lucid. The remaining authors disclose no conflicts.

Funding:
T32-DK-007740; Consortium of Eosinophilic Gastrointestinal Disease Researchers (CEGIR) Hirano Training Award (U54AI117804); L30-DK144931; All work was independent of the funding.

Ethical Statement:
This study was approved by the University of Pennsylvania Institutional Review Board. The requirement for informed consent was waived due to the retrospective and deidentified nature of the study.

Data Transparency Statement:
The data underlying this study are available from the corresponding author upon reasonable request and completion of appropriate data use agreements. The analytic code and programming scripts used for this analysis will also be made available to qualified researchers to facilitate reproducibility. This study was not registered and had no public involvement.

Reporting Guidelines:
TRIPOD-LLM.

Gastro Hep Advances, Volume 5

Supplemental information

Development and Validation of a Large Language Model Case Identification Strategy for Eosinophilic Esophagitis

Corey J. Ketchem, Uğurcan Vurgun, Agnes Wang, Sunil Thomas, Ashley Batugo, John E. Pandolfino, Gary W. Falk, Kristle L. Lynch, Evan S. Dellon, Danielle L. Mowery, and James D. Lewis

Supplemental Tables and Figures

Supplemental Table 1. Definitions of the 10 variables extracted from the relevant clinical and histopathologic text.

Variable (name)	Definition
Positive eosinophils (“eos_present”)	Indicates whether eosinophils are positively mentioned in any part of the biopsy report; answer “No” if explicitly stated as absent throughout (“no eosinophils”) or no mention of eosinophils.
Eosinophils located in the esophagus (“eos_location___esoph”)	Specifies if eosinophils are detected in esophageal tissue; answer “No” if they are absent, unclear, or only described in other anatomic locations.
Eosinophils enumerated (“eos_count”)	Indicates whether a specific numeric eosinophil count per high-power field (eos/hpf) is reported in the biopsy report.
Peak eosinophil count (“esoph_eos_num”)	The highest reported eosinophil count (per high-power field) in any esophageal biopsy section. If multiple values are present, select the maximum; if no numeric value is given for the esophagus, enter “0”.
Descriptive increase of eosinophils (“esoph_eos_num_desc_increased”)	Indicates whether there is descriptive language in the esophageal biopsy suggesting increased eosinophils, including terms like “increased”, “numerous”, or “eosinophilic infiltration”.
Dysphagia (“dysphagia”)	Presence of dysphagia symptoms (trouble swallowing, food sticking, etc.) in the clinical report. Must be non-negated. Historic or intermittent symptoms qualify.
Food impation (“food_impact”)	Indicates whether there is mention of food impaction (food getting stuck or obstructing swallowing), which is more severe than dysphagia alone. Requires explicit or synonymous terminology.
Atypical esophageal symptoms (“reflux”)	Presence of reflux, heartburn, regurgitation or related diagnosis noted in the clinical report.
Documented past EoE history (“pmh_eoe”)	Whether the patient has a prior diagnosis or history of eosinophilic esophagitis (EoE) listed in the clinical report. Mention must refer to established history, not just differential.
EoE diagnosis (“eoe_dx”)	Indicates if the patient meets criteria for a diagnosis of EoE based on a combination of biopsy and clinical report findings (i.e., increased eosinophils in the esophagus and relevant symptoms, or documented history of EoE).

Supplemental Figure 1. Human-in-the-loop process for development of large language model (LLM) pipeline to extract clinical variables from unstructured text.

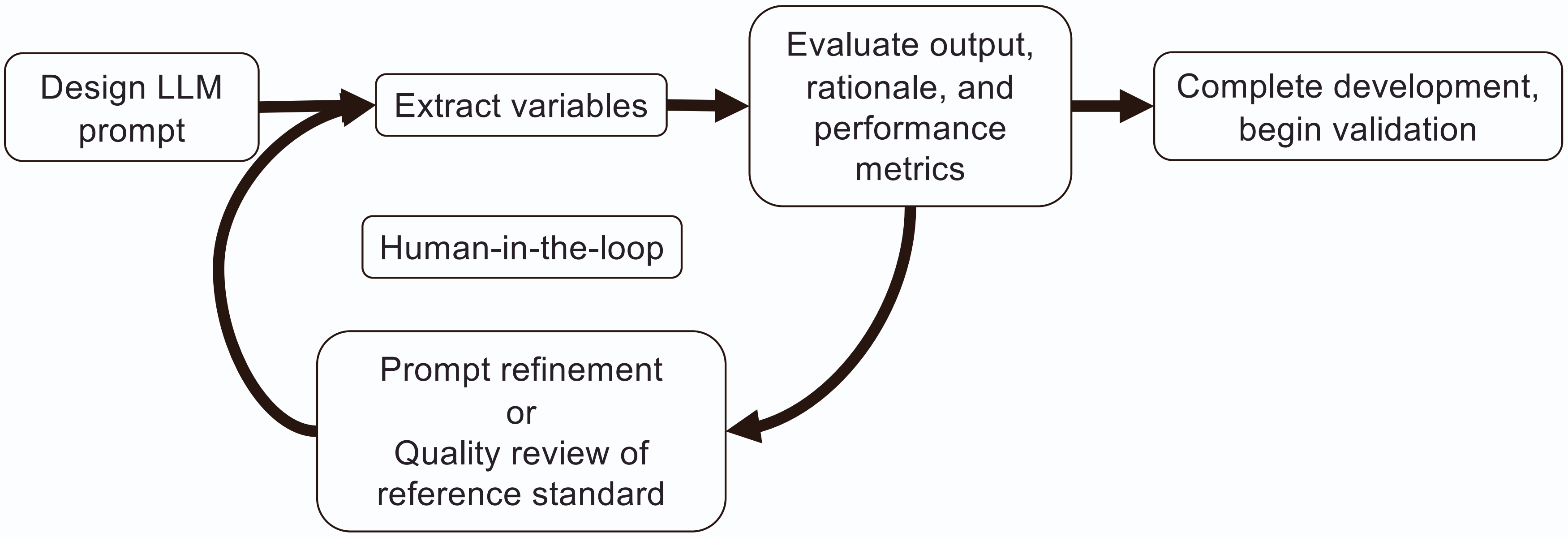

Supplemental Table 2. Rules-based NLP development and validation performance metrics, point estimates with 95% confidence intervals.

Method	Precision (PPV)	Recall (Sensitivity)	Specificity	NPV	Accuracy	Kappa	F1
**Development Set**							
**ICD alone**	0.86 [0.76,0.96]	0.75 [0.64,0.86]	0.95 [0.92,0.99]	0.91 [0.86,0.95]	0.90 [0.85,0.94]	0.73 [0.62,0.84]	0.80 [0.71,0.89]
**RB-NLP diagnosis**	0.72 [0.61,0.82]	0.86 [0.77,0.95]	0.87 [0.81,0.92]	0.94 [0.90,0.98]	0.87 [0.82,0.91]	0.68 [0.57,0.80]	0.78 [0.69,0.87]
**ICD+RB diagnosis**	0.71 [0.60,0.81]	0.95 [0.89,1.0]	0.85 [0.79,0.91]	0.98 [0.95,1.0]	0.88 [0.83,0.92]	0.72 [0.62,0.82]	0.81 [0.73,0.89]
**Validation Set**							
**ICD alone**	0.97 [0.91,1.0]	0.86 [0.75,0.97]	0.98 [0.95,1.0]	0.93 [0.86,1.0]	0.94 [0.89,1.0]	0.87 [0.76,0.97]	0.91 [0.84,1.0]
**RB-NLP diagnosis**	0.75 [0.61,0.89]	0.75 [0.61,0.89]	0.86 [0.77,0.95]	0.86 [0.77,0.95]	0.82 [0.75,0.90]	0.61 [0.45,0.77]	0.75 [0.62,0.88]
**ICD+RB diagnosis**	0.78 [0.65,0.91]	0.89 [0.79,0.99]	0.86 [0.77,0.95]	0.93 [0.87,1.0]	0.87 [0.80,0.94]	0.73 [0.59,0.86]	0.83 [0.73,0.93]

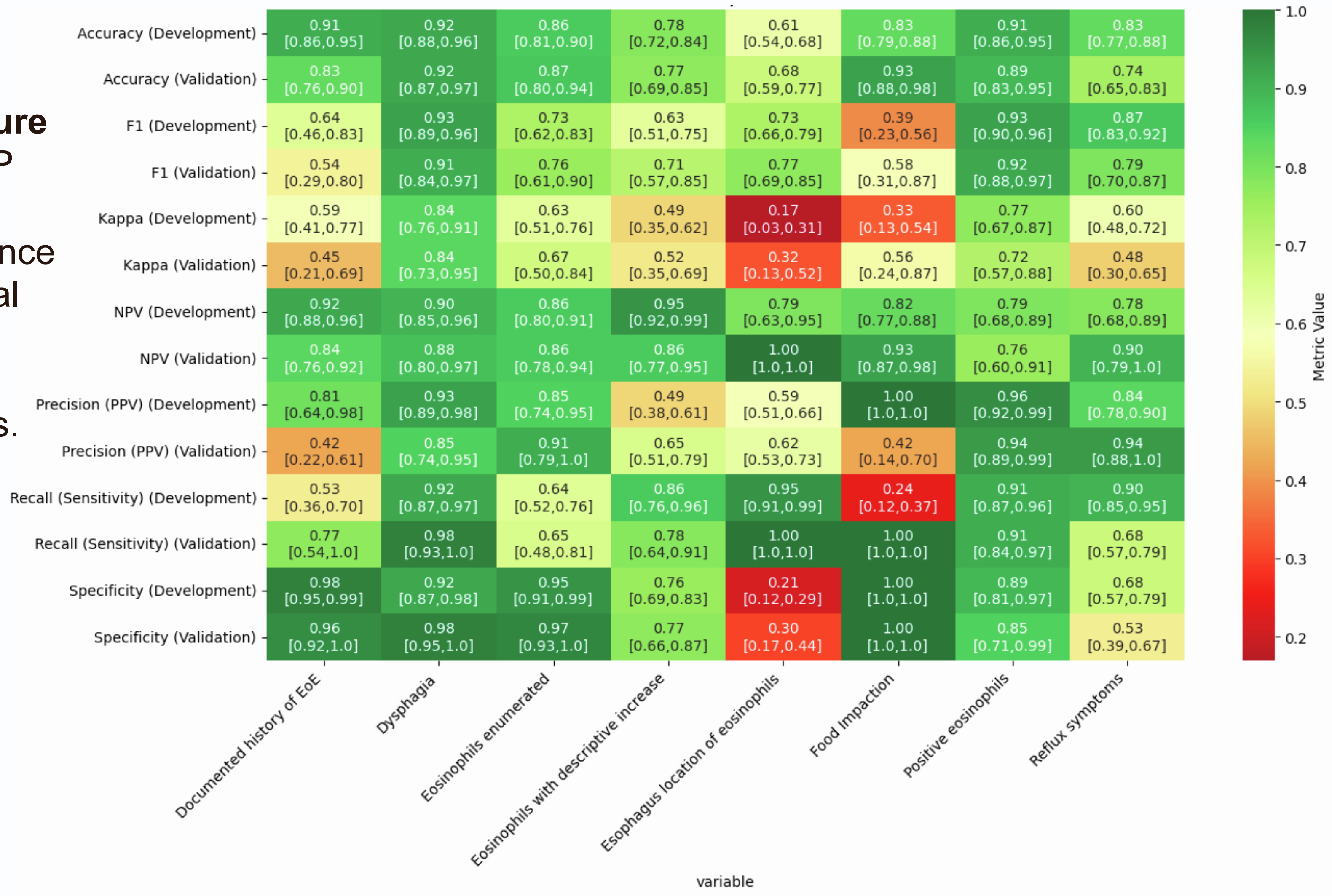

Supplemental Figure 2. Rules-based NLP development and validation performance metrics for individual variables, point estimates with 95% confidence intervals.

Supplemental Table 3. Demographics and clinical characteristics of the LLM-first cohort. Histology and symptom features are LLM-NLP assigned.

	LLM-first validation (N=93)
**Demographics, n(%)**	
Age at biopsy	46.5 (35.0-62.0)
Female	60 (60)
Male	40 (40)
**Race, n(%)**	
Asian	3 (3)
Black or African American	11 (11)
Multiracial	1 (1)
Patient Declined	1 (1)
Some Other Race	3 (3)
Unknown	2 (2)
White	79 (79)
**Ethnicity, n(%)**	
Hispanic Latino	2 (2)
Not Hispanic or Latino	97 (97)
**Histology, n(%)**	
Positive eosinophils	47 (47)
Eosinophils located in esophagus	42 (42)
Eosinophil counts increased (≥15 eos/hpf)	31 (31)
Eosinophil count	30.0 (6.0-50.0)
**Symptoms and history, n(%)**	
Dysphagia	50 (50)
Food impaction	18 (18)
Reflux symptoms	59 (59)
Documented past EoE history	15 (15)
Manually annotated EoE diagnosis	29 (29)
EoE diagnostic code	34 (34)

Supplemental Table 4. LLM-NLP-first development and validation performance metrics, point estimates with 95% confidence intervals.

Method	Precision (PPV)	Recall (Sensitivity)	Specificity	NPV	Accuracy	Kappa	F1
ICD	0.90 [0.79,1.0]	0.90 [0.79,1.00]	0.95 [0.90,1.0]	0.95 [0.90,1.0]	0.94 [0.89,0.99]	0.85 [0.73,0.97]	0.90 [0.81,0.98]
LLM-derived features	0.93 [0.83,1.0]	0.90 [0.79,1.0]	0.97 [0.93,1.0]	0.95 [0.90,1.0]	0.95 [0.90,0.99]	0.87 [0.77,0.98]	0.91 [0.83,0.99]
LLM-assigned diagnosis	1.0 [1.0,1.0]	0.79 [0.65,0.94]	1.0 [1.0,1.0]	0.91 [0.85,0.98]	0.94 [0.89,0.99]	0.84 [0.72,0.96]	0.89 [0.79,0.98]
ICD+LLM diagnosis	0.90 [0.79,1.0]	0.93 [0.84,1.0]	0.95 [0.90,1.0]	0.97 [0.93,1.0]	0.95 [0.90,0.99]	0.91 [0.83,1.0]	0.92 [0.84,0.99]

Supplemental Figure 3. Scatter plots comparing eosinophil counts extracted by the rules-based NLP pipeline to manually annotated reference-standard counts in esophageal biopsy reports for both (A) RB-NLP validation and (B) LLM-NLP validation. The blue line indicates the best-fit linear regression.

A.

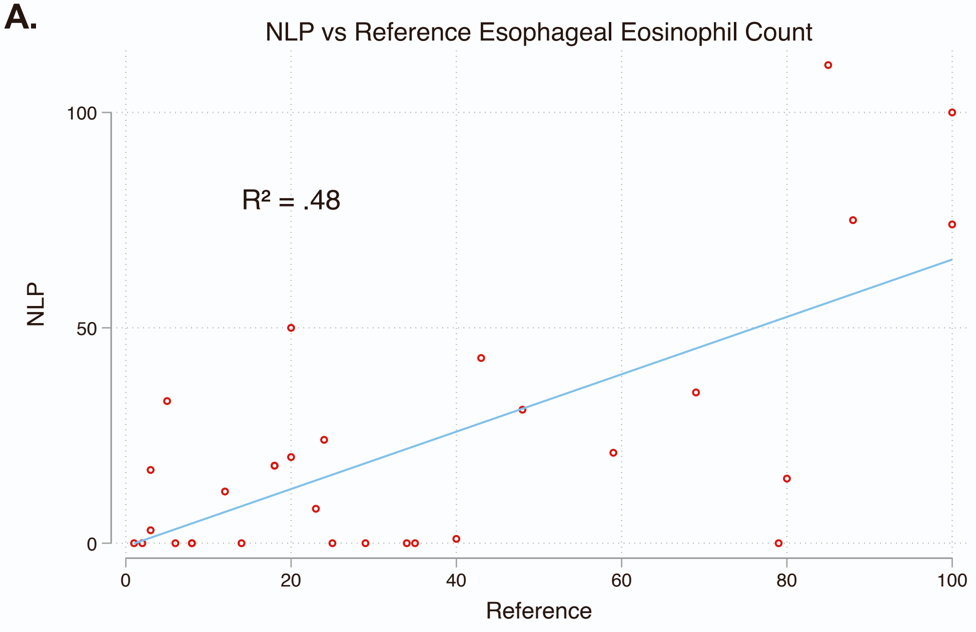

B.

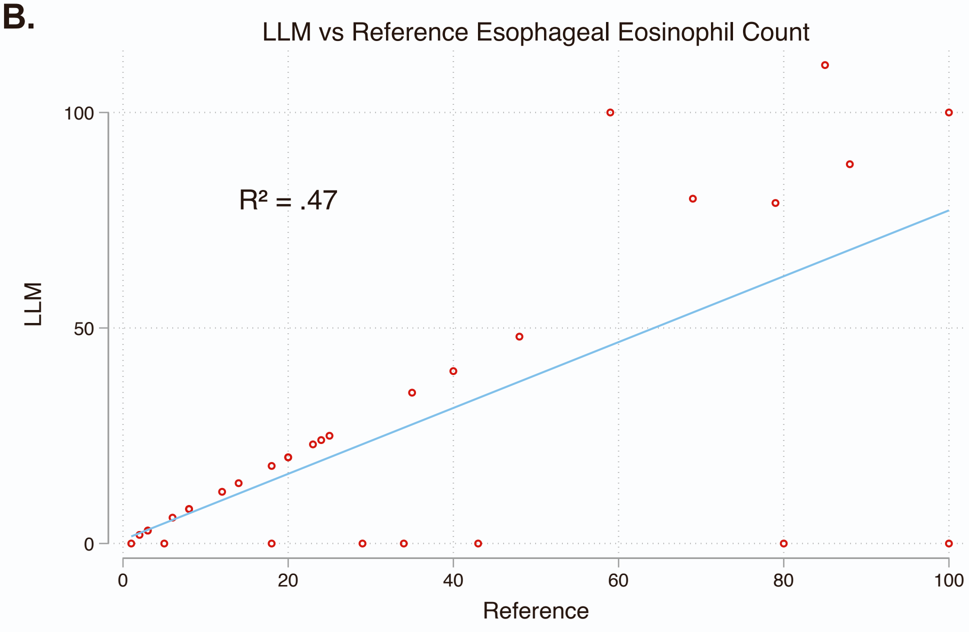

SUPPLMENTAL METHODS

To develop the cohort, we queried Penn Medicine patients with clinical encounters from 2008 through April 2024 with patients initially identified by surgical pathology procedure orders (Figure 1). The Penn Medicine system includes the Hospital of the University of Pennsylvania, Pennsylvania Hospital, Penn Presbyterian Medical Center, and several smaller primarily outpatient facilities. For NLP pipeline development and validation, we included patients with at least one associated clinical note occurring between 1 and 60 days prior to the corresponding biopsy date to ensure extracted clinical information reflected the pre-biopsy context. This filtering narrowed the calendar years of cases between 2015 through April 2024. From a sample of 1,000 patients meeting these criteria, we randomly allocated them to a development cohort (n=200; 56 with EoE) and a validation cohort (n=100; 36 with EoE). This approach allowed for a temporally-aligned, filtered cohort suitable for downstream NLP and analysis. Assuming a PPV of 80%, the sample sizes yielded 95% confidence intervals with margins of error ranging from ±9% to ±11%. Due to token (smaller fundamental units of text) limitations, the first clinic and biopsy notes per patient were analyzed during the NLP tasks. Only observations with complete data were used. Manual chart review (performed by CJK, gastroenterologist) of the deidentified clinic note and biopsy report was used to assign the reference standard for key clinical variables and EoE diagnosis (symptoms + positive histology or history of EoE) in both development and validation cohorts (Supplemental Table 1).

RB-NLP Pipeline

RB-NLP was constructed on top of spaCy with medSpaCy's clinical components enabled. For sentence segmentation, we applied spaCy's sentencizer preceded by a custom boundary-setting component to improve segmentation accuracy around bullet points and section headers. For entity extraction, we curated a set of TargetRule definitions using medSpaCy's TargetMatcher to identify key clinical concepts relevant to EoE (e.g., "trouble swallowing" → dysphagia). These rules produced binary features indicating whether each concept was present anywhere in the document (Supplemental Table 1). Eosinophil counts per high-power field (HPF) were extracted using regular expressions. Because descriptive eosinophil terminology can appear outside structured count statements, we manually evaluated these descriptors and found that phrases indicating increased eosinophils corresponded to a median peak of 35 eos/HPF (IQR 20.5–51.5). Accordingly, these descriptions were operationalized as meeting the ≥15 eos/HPF threshold for EoE. For assertion detection, we incorporated

medSpaCy's ConText component to determine whether extracted entities were negated ("no eosinophils"), historical ("history of EoE"), or modified by intensity or temporality.

Diagnostic decision logic for both the reference standard (human applied) and NLP approaches:

- A patient was classified as EoE if either
    1. a historical diagnosis of EoE was identified in the clinic note (not negated), or
    2. pathology features extracted from the pathology report met EoE histologic criteria (≥15 eos/HPF or descriptive increases mapped to this threshold) AND features extracted from the clinic note met EoE symptom criteria .
- All extracted features were evaluated at the document level; if any qualifying feature appeared in the note and was not negated or historical-only, it contributed to the classification.
- No machine-learning classifier was used; classification relied entirely on deterministic rule-based thresholds.

RB-NLP predictions were compared with the manually annotated reference standard using binary classification metrics. The programming code for the RB-NLP pipeline is available upon request.

LLM-NLP Pipeline

For the LLM portion of the project, we followed TRIPOD-LLM reporting guidelines.(1) During prompt development, we used established clinical criteria and domain knowledge relevant to EoE diagnosis. Utilizing human-in-the-loop iterative refinement process, we aimed to address several failure modes encountered during early testing (Supplemental Figure 1).(2) These included (i) misinterpretation of negated or ambiguous phrases, (ii) difficulty distinguishing historical from current diagnoses, and (iii) inconsistent recognition of eosinophil descriptors that lacked explicit numerical counts (e.g., "increased eosinophils," "numerous eosinophils"). To mitigate these issues, we structured the prompt around established EoE diagnostic criteria and incorporated balanced positive and negative examples for each binary field, including common negations and linguistically ambiguous terms ("rare eosinophils," "negative for eosinophils"). This design was motivated by the need to

ensure consistent interpretation of subtle clinical language and to reduce hallucinated or unsupported extractions.

Source text was segmented by section headers (e.g., BIOPSY REPORT, CLINICAL REPORT) to help the model correctly anchor eosinophil counts and diagnostic statements in their proper clinical context. Numeric eosinophil values were extracted using deterministic rules but verified by the LLM to address transcription-like inconsistencies across notes. We also required the model to provide textual evidence and a brief rationale for each output to promote transparency, allow interrogation of failure cases, and streamline quality assessment.

After each iteration, LLM outputs, rationales, and misclassified examples were reviewed to determine whether errors were attributable to prompt limitations or reference standard issues. Refinement continued until the LLM-assigned diagnosis exceeded the F1-score of ICD alone. A temperature of 0.2 was used throughout development to improve determinism. Once performance stabilized and no further failure modes emerged, model development was concluded.

Additional LLM-NLP Validation in Unannotated Cohorts

Using the remaining unannotated and unseen cases, we evaluated the performance of an LLM-first labeling approach. The LLM-NLP pipeline was applied prior to assigning the reference standard, allowing retrospective validation with knowledge of outputs. This strategy enables assessment of LLM-NLP scalability compared to a manual annotation-first approach. We measured performance metrics for the LLM-assigned diagnosis of EoE on 93 cases (7 removed for inadequate documentation). Further, we applied the pipeline to 580 cases to quantify the number of EoE cases identified with ICD alone, LLM-NLP alone, and LLM-ICD combination. Demographics and LLM-NLP derived clinical features were compared by case identification strategies.

Performance Metric Definitions (Lay Explanation)

To aid interpretation of model performance, we provide simplified explanations of the evaluation metrics reported in this study:

- Accuracy: The proportion of all predictions the model gets right.

    - Example: How often the model correctly identifies whether a patient does or does not have EoE.
- Precision (Positive Predictive Value, PPV): When the model predicts that a patient has EoE, how often that prediction is correct.
    - Example: “Of all the patients the model labeled as EoE, how many truly had the condition?”
- Recall (Sensitivity): How well the model finds true cases.
    - Example: “Of all patients who truly had EoE, how many did the model successfully identify?”
- Specificity: How well the model avoids false alarms.
    - Example: “Of all patients who did NOT have EoE, how many did the model correctly identify as not having it?”
- F1 Score: A combined measure of precision and recall, balancing how well the model avoids false positives and how well it captures true positives.
    - Example: A single number that summarizes both “how many true cases the model finds” and “how often those identifications are correct.”

Structured information extraction prompt for LLM-NLP pipeline to identify key EoE variables.

**Finalized Prompt**
You are a specialized medical information extraction system with expertise in gastroenterology and pathology, with a focus on eosinophilic gastrointestinal diseases (EGIDs), including eosinophilic esophagitis (EoE)<br>TASK: Extract specific structured information from the following medical note about eosinophils, EGID diagnosis, and related symptoms. Read the ENTIRE note thoroughly before answering.<br>CLINICAL AND BIOPSY REPORT:<br>{text}<br>EXTRACTION GUIDELINES:<br>1. Eosinophils Present (eos_present):<br>- Only evaluate the text in the BIOPSY REPORT. Ignore all text that appears before this section.<br>- Answer "Yes" if ANY of these conditions are met:<br>* There is a positive mention of the terms: "eosinophil(s)", "intraepithelial eosinophils", "eosinophilic", or "eosinophilia", without a negation.<br>* Positive descriptive qualifiers include "few", "rare", "scattered", "present", "increased", or "prominent" eosinophils.<br>- Answer "No" ONLY if:<br>* The report explicitly states absence of eosinophils using terms like: "no eosinophils", "no evidence of", "absent", "not seen", "negative for eosinophils, “no increase in intraepithelial eosinophils”, or similar phrases indicating lack of histologic increase.<br>- IMPORTANT:<br>* If eosinophils are mentioned with any non-negated descriptor, even if minimal (e.g., “rare eosinophils present”), answer “Yes”.<br>* Be cautious with negation terms near “eosinophil” mentions. A negated context (e.g., “no eosinophils seen”) should be interpreted as “No”.<br>* Mentions of blood eosinophil levels (e.g., “eosinophil count in blood”, “peripheral eosinophilia”) from a CBC are not considered evidence of eosinophils in the biopsy.<br>2. Eosinophils in Esophagus (eos_location___esoph):<br>- Only evaluate the text in the BIOPSY REPORT. Ignore all content before this section.<br>- Answer "Yes" if ANY of these conditions are met:<br>* Eosinophils are described in a section clearly labeled or referring to esophageal tissue, including (but not limited to) the following headers or phrases: "Esophagus, biopsy:", "Esophagus:", "Mid esophagus", "Distal esophagus", "Proximal esophagus", "Gastroesophageal", "Esophagogastric", or "Esophageal-gastric junction"<br>* There is a mention of eosinophil-related findings (e.g., "increased eosinophils", "eosinophilic infiltrate", "rare eosinophils", "eosinophil count", or "increased intraepithelial eosinophils") within or immediately following an esophageal section, with no intervening section header indicating a different anatomical site.<br>- Answer "No" if:<br>* Eosinophils are only described in non-esophageal locations, such as the stomach, duodenum, colon, ileum, or any other labeled section not referring to the esophagus.<br>* Eosinophils are explicitly stated as absent, not seen, no evidence, or negative in the esophageal section.

     * The anatomical location where eosinophils are observed is unclear or cannot be confidently attributed to the esophagus.
     * There is no mention of eosinophils in association with esophageal tissue.
   - IMPORTANT:
     * Use section headers as anchors to assign findings to a specific location.
     * When eosinophils are described after an "Esophagus" header, assume they apply to that location unless a new header appears.
     * Mentions of eosinophils in blood or systemic references (e.g., “peripheral eosinophilia”) should be ignored.
3. Eosinophil Count Provided (eos_count):
   - Only evaluate the text in the BIOPSY REPORT. Ignore all text that appears before this section.
   - Answer "Yes" if ANY of these conditions are met in the biopsy report:
     * ANY specific number or range of eosinophils is mentioned (e.g., “15 eosinophils per high power field”, “15 eos/HPF”, “15 eos/hpf”, “15/hpf”, “20–30 eosinophils per HPF”)
     * A numeric threshold is provided (e.g., "eosinophils >15/HPF", "less than 5 eos/hpf")
     * A count of zero is numerically specified (e.g., “0 eosinophils/HPF” or “no eosinophils (0/HPF)”
     * The eosinophil count appears in a section labeled as or clearly referring to the esophagus (e.g., "Esophagus", "Mid esophagus", etc.).
   - Answer "No" if:
     * Only qualitative descriptions are given (e.g., "few eosinophils", "many", "prominent", "increased", "rare"), without a numeric value.
     * No specific numeric value related to eosinophils appears anywhere in the biopsy report section.
4. Esophageal Eosinophil Number (esoph_eos_num):
   - Only evaluate the text in the BIOPSY REPORT. Ignore all text that appears before this section.
   - In the biopsy report, Identify all numeric eosinophil counts per HPF reported for esophageal locations, and extract the single highest value.
     * For a single value (e.g., "40 eos/HPF"), report just "40"
     * For a range (e.g., "0-1 eos/HPF"), report the "1"
     * For a threshold (e.g., ">15 eos/HPF"), report "15"
     * For multiple counts from different locations in the esophagus, report the HIGHEST value
   - Report "0" if no specific eosinophil count is provided for the esophagus
   - Examples of correct extraction:
     * From "20 eosinophils per high-power field" ? "20"
     * From "eosinophil counts ranging from 5-30/HPF" ? "30"
     * From "proximal: 10 eos/HPF, distal: 45 eos/HPF" ? "45"
5. Increased Eosinophil Description (esoph_eos_num_desc_increased):
   - Only evaluate the text in the BIOPSY REPORT. Ignore all text that appears before this section.
   - Answer "Yes" if ANY of these conditions are met:
     *The biopsy report includes descriptive language indicating increased or elevated eosinophils in the esophagus, such as: "increased eosinophils", "elevated eosinophils", "numerous eosinophils", "many eosinophils", "marked eosinophilia", "dense eosinophilic infiltrate", "eosinophil-rich inflammation"
     * The pathologist implies increased eosinophilic activity or disease even without using the word "increased", such as: "eosinophilic infiltration", "expansion of eosinophils", "active esophagitis with eosinophils"
     * Pathologist interpretation suggesting increased eosinophils even without the explicit term "increased"
     * A mention of "active" disease
   - Answer "No" if:
     * Eosinophils are described as "rare", "few", "occasional", "scant", or "normal"
     * Only a numeric count is given without qualitative description
     * No qualitative assessment of eosinophil quantity is provided
   - NOTE: If both a qualitative description AND a numeric count are given, still answer "Yes" if the description indicates increase
6. Dysphagia (dysphagia):
- Focus on the CLINICAL REPORT
   - Answer "Yes" if ANY of these conditions are met:
     * The term "dysphagia" appears and is not negated
     * Patient reports difficulty swallowing or food getting stuck
     * Descriptions of "trouble swallowing", "choking sensation", “food moving slowly”, or "food sticking" in throat/chest
   - Answer "No" if:
     * Such symptoms are explicitly denied (e.g., "no dysphagia", "denies difficulty swallowing")
     * There is no mention of swallowing difficulties
   - IMPORTANT: Even historic or intermittent dysphagia should be marked "Yes"
7. Food Impaction (food_impact):
- Focus on the CLINICAL REPORT
   - Answer "Yes" if ANY of these conditions are met:
     * Explicit mention of "food impaction" or "food bolus obstruction"
     * Descriptions of food or bolus getting stuck
     * Patient reporting "food or liquids won't go down" or "complete blockage"
     * History of endoscopy to remove food impaction
     * Terms like "impaction", "obstruction", or "sticking" in relation to food
   - Answer "No" if:
     * Only mild dysphagia without complete obstruction is reported
     * No mention of food getting stuck
     * Food-related symptoms are explicitly denied
   - NOTE: Food impaction is more severe than dysphagia alone; it involves food getting completely stuck
8. Reflux (reflux):
- Focus on the CLINICAL REPORT
- Answer "Yes" if ANY of these conditions are met:
     * Direct mention of reflux symptoms, such as "reflux", "GERD", “burning in chest”, “heartburn”, “regurgitation”, or "gastroesophageal reflux"
     * Past medical history of reflux
     * Medications with an indication to treat reflux only
   - Answer "No" if:
     * Reflux is explicitly denied (e.g., "no reflux symptoms", "denies GERD")
     * No mention of reflux or GERD anywhere in the note
9. Past Medical History of EoE (pmh_eoe):
- Focus on the CLINICAL REPORT

- Answer "Yes" if ANY of these conditions are met:
  * The clinical report states a past medical history of "eosinophilic esophagitis", "EoE", "esophageal eosinophilia", or "PPI-responsive esophageal eosinophilia".
  * Terms like "known EoE", "established EoE", "history of EoE", "previously diagnosed with EoE", or "#Eosinophilic esophagitis"
  * EoE listed under "Past Medical History" or "PMH" sections
- Answer "No" if:
  * EoE is mentioned only as a differential diagnosis
  * No indication of a pre-existing EoE diagnosis before current encounter

10. EoE Diagnosis (eoe_dx):
- Use information from both the CLINICAL REPORT and the BIOPSY REPORT to determine whether the patient meets criteria for eosinophilic esophagitis (EoE).
- Answer "Yes" if either of the following is true:
  * The clinical report states a past medical history of "eosinophilic esophagitis", "EoE", "esophageal eosinophilia", or "PPI-responsive esophageal eosinophilia".
  * The BIOPSY REPORT has a descriptive increase in eosinophils in the esophagus (e.g., “mildly increased,” “moderately increased,” or “increased”) OR a numerical count ≥15 eosinophils per high power field (eos/hpf) AND the CLINICAL REPORT includes symptoms consistent with EoE, such as dysphagia, food impaction, chest pain, heartburn, or regurgitation.
- If none of the above criteria are met, answer "No".

IMPORTANT CONTEXT:
In pathology reports, the following findings often indicate EoE:
- Basal zone hyperplasia
- Dilated intercellular spaces (spongiosis)
- Subepithelial fibrosis
- Surface layering of eosinophils or eosinophil microabscesses

RESPONSE FORMAT:
For each field, provide your answer AND a brief rationale citing direct evidence from the note:
eos_present: Yes/No
Rationale: [Cite specific text and explain your reasoning]
eos_location___esoph: Yes/No
Rationale: [Cite specific text and explain your reasoning]
eos_count: Yes/No
Rationale: [Cite specific text and explain your reasoning]
esoph_eos_num: [exact number or "0"]
Rationale: [Cite specific text and explain your reasoning]
esoph_eos_num_desc_increased: Yes/No
Rationale: [Cite specific text and explain your reasoning]
dysphagia: Yes/No
Rationale: [Cite specific text and explain your reasoning]
food_impact: Yes/No
Rationale: [Cite specific text and explain your reasoning]
reflux: Yes/No
Rationale: [Cite specific text and explain your reasoning]
pmh_eoe: Yes/No
Rationale: [Cite specific text and explain your reasoning]
eoe_dx: Yes/No
Rationale: [Cite specific text and explain your reasoning]
YOU MUST PROVIDE A DETAILED RATIONALE FOR EACH FIELD, EVEN IF THE ANSWER IS "NO" OR "0".

REFERENCES

1. Gallifant J, Afshar M, Ameen S, et al. The TRIPOD-LLM reporting guideline for studies using large language models. Nat Med 2025;31:60-69.

2. Wang ZJ, Choi D, Xu S, et al. Putting humans in the natural language processing loop: A survey. arXiv preprint arXiv:2103.04044 2021.

The TRIPOD-LLM Statement: A Targeted Guideline For Reporting Large Language Models Use

Supplementary Table 2: Fillable TRIPOD-LLM checklist

Section	Item	Checklist Item	Research Design	LLM Task	Page
Title					
Title	1	Identify the study as developing, fine-tuning, and/or evaluating the performance of an LLM, specifying the task, the target population, and the outcome to be predicted.	All	All	4, 5
Abstract					
Abstract	2	See TRIPOD-LLM for Abstracts	All	All	
Introduction					
Background	3a	Explain the healthcare context / use case (e.g., administrative, diagnostic, therapeutic, clinical workflow) and rationale for developing or evaluating the LLM, including references to existing approaches and models.	All	All	4
	3b	Describe the target population and the intended use of the LLM in the context of the care pathway, including its intended users in current gold standard practices (e.g., healthcare professionals, patients, public, or administrators).	E<br>H	All	4
Objectives	4	Specify the study objectives, including whether the study describes the initial development, fine-tuning, or validation of an LLM (or multiple stages).	All	All	4
Methods					
Data	5a	Describe the sources of data separately for the training, tuning, and/or evaluation datasets and the rationale for using these data (e.g., web corpora, clinical research/trial data, EHR data, or unknown).	All	All	5, Supplemental
	5b	Describe the relevant data points and provide a quantitative and qualitative description of their distribution and other relevant descriptors of the dataset (e.g., source, languages, countries of origin)	All	All	5, Supplemental
	5c	Specifically state the date of the oldest and newest item of text used in the development process (training, fine-tuning, reward modeling) and in the evaluation datasets.	All	All	5, Supplemental
	5d	Describe any data pre-processing and quality checking, including whether this was similar across text corpora, institutions, and relevant socio-demographic groups.	All	All	Supplement
	5e	Describe how missing and imbalanced data were handled and provide reasons for omitting any data.	All	All	Supplement
Analytical Methods	6a	Report the LLM name, version, and last date of training.	All	All	6
	6b	Report details of LLM development process, such as LLM architecture, training, fine-tuning procedures, and alignment	M<br>D	All	NA

		strategy (e.g., reinforcement learning, direct preference optimization, etc.) and alignment goals (e.g., helpfulness, honesty, harmlessness, etc.).			
	6c	Report details of how text was generated using the LLM, including any prompt engineering (including consistency of outputs), and inference settings (e.g., seed, temperature, max token length, penalties), as relevant.	M<br>D<br>E	All	Supplemental
	6d	Specify the initial and post-processed output of the LLM (e.g., probabilities, classification, unstructured text).	All	All	Supplemental
	6e	Provide details and rationale for any classification and, if applicable, how the probabilities were determined and thresholds identified.	All	C<br>OF	NA
LLM Output	7a	Include metrics that capture the quality of generative outputs, such as consistency, relevance, and accuracy, compared to gold standards.	All	QA<br>IR<br>DG<br>SS<br>MT	7, 8
	7b	Report the outcome metrics' relevance to downstream task at deployment time and, where applicable, correlation of metric to human evaluation of the text for the intended use.	E<br>H	All	7, 8
	7c	Clearly define the outcome, how the LLM predictions were calculated (e.g., formula, code, object, API), the date of inference for closed-source LLMs, and evaluation metrics.	E<br>H	All	Supplemental
	7d	If outcome assessment requires subjective interpretation, describe the qualifications of the assessors, any instructions provided, relevant information on demographics of the assessors, and inter-assessor agreement.	All	All	Supplemental
	7e	Specify how performance was compared to other LLMs, humans, and other benchmarks or standards.	All	All	Supplemental
Annotation	8a	If annotation was done, report how text was labeled, including providing specific annotation guidelines with examples.	All	All	Supplemental
	8b	If annotation was done, report how many annotators labeled the dataset(s), including the proportion of data in each dataset that were annotated by more than 1 annotator, and the inter-annotator agreement.	All	All	Supplemental
	8c	If annotation was done, provide information on the background and experience of the annotators or characteristics of any models involved in labelling.	All	All	Supplemental
Prompting	9a	If research involved prompting LLMs, provide details on the processes used during prompt design, curation, and selection.	All	All	Supplemental
	9b	If research involved prompting LLMs, report what data were used to develop the prompts.	All	All	Supplemental
Summarization	10	Describe any preprocessing of the data before summarization.	All	SS	Supplemental
Instruction tuning/Alig	11	If instruction tuning/alignment strategies were used, what were the instructions, data, and interface used for evaluation,	M<br>D	All	NA

nment		and what were the characteristics of the populations doing evaluation?			
Compute	12	Report compute, or proxies thereof (e.g., time on what and how many machines, cost on what and how many machines, inference time, floating-point operations per second (FLOPs)), required to carry out methods.	M D E	All	6
Ethical Approval	13	Name the institutional research board or ethics committee that approved the study and describe the participant-informed consent or the ethics committee waiver of informed consent.	All	All	6
Open Science	14a	Give the source of funding and the role of the funders for the present study.	All	All	2
	14b	Declare any conflicts of interest and financial disclosures for all authors.	All	All	2
	14c	Indicate where the study protocol can be accessed or state that a protocol was not prepared.	H	All	1
	14d	Provide registration information for the study, including register name and registration number, or state that the study was not registered.	H	All	1
	14e	Provide details of the availability of the study data.	All	All	1
	14f	Provide details of the availability of the code to reproduce the study results.	All	All	1
Public Involvement	15	Provide details of any patient and public involvement during the design, conduct, reporting, interpretation, or dissemination of the study or state no involvement.	H	All	1
Results					
Participants	16a	When using patient/EHR data, describe the flow of text/EHR/patient data through the study, including the number of documents/questions/participants with and without the outcome/label and follow-up time as applicable.	E H	All	Supplemental
	16b	When using patient/EHR data, report the characteristics overall and, for each data source or setting, and for development/evaluation splits, including the key dates, key characteristics, and sample size.	E H	All	Supplemental
	16c	For LLM evaluation that include clinical outcomes, show a comparison of the distribution of important clinical variables that may be associated with the outcome between development and evaluation data, if available.	E H	All	Table 1
	16d	When using patient/EHR data, specify the number of participants and outcome events in each analysis (e.g., for LLM development, hyperparameter tuning, LLM evaluation).	E H	All	Table 1
Performance	17	Report LLM performance according to pre-specified metrics (see item 7a) and/or human evaluation (see item 7d).	All	All	Table 3
LLM Updating	18	If applicable, report the results from any LLM updating, including the updated LLM and subsequent performance.	All	All	NA

Discussion					
Interpretation	19a	Give an overall interpretation of the main results, including issues of fairness in the context of the objectives and previous studies.	All	All	9-12
Limitations	19b	Discuss any limitations of the study and their effects on any biases, statistical uncertainty, and generalizability.	All	All	11
Usability of the LLM in context	19c	Describe any known challenges in using data for the specified task and domain context with reference to representation, missingness, harmonization, and bias.	E H	All	11
	19d	Define the intended use for the implementation under evaluation, including the intended input, end-user, level of autonomy/human oversight.	E H	All	9-12
	19e	If applicable, describe how poor quality or unavailable input data should be assessed and handled when implementing the LLM, i.e., what is the usability of the LLM in the context of current clinical care.	E H	All	11
	19f	If applicable, specify whether users will be required to interact in the handling of the input data or use of the LLM, and what level of expertise is required of users.	E H	All	11
	19g	Discuss any next steps for future research, with a specific view to applicability and generalizability of the LLM.	All	All	9-12

LLM = large language model; M = LLM methods; D = *de novo* LLM development; E = LLM evaluation; H = LLM evaluation in healthcare settings; C = classification; OF = outcome forecasting; QA = long-form question-answering; IR = information retrieval; DG = document generation; SS = summarization and simplification; MT = machine translation; EHR = electronic health record.

Note: For studies using existing LLMs, users should include reference(s) to reportable information if provided by the original developers or state that this information is not available.

Table 3
Table 3
